# HapX Positively and Negatively Regulates the Transcriptional Response to Iron Deprivation in *Cryptococcus neoformans*

**DOI:** 10.1371/journal.ppat.1001209

**Published:** 2010-11-24

**Authors:** Won Hee Jung, Sanjay Saikia, Guanggan Hu, Joyce Wang, Carlen Ka-Yin Fung, Cletus D'Souza, Rick White, James W. Kronstad

**Affiliations:** 1 Department of Biotechnology, Chung-Ang University, Daedeok-Myeon, Anseong-Si, Gyeonggi-Do, Republic of Korea; 2 The Michael Smith Laboratories, Department of Microbiology and Immunology, and Faculty of Land and Food Systems, University of British Columbia, Vancouver, British Columbia, Canada; 3 Department of Statistics, University of British Columbia, Vancouver, British Columbia, Canada; University of Melbourne, Australia

## Abstract

The fungal pathogen *Cryptococcus neoformans* is a major cause of illness in immunocompromised individuals such as AIDS patients. The ability of the fungus to acquire nutrients during proliferation in host tissue and the ability to elaborate a polysaccharide capsule are critical determinants of disease outcome. We previously showed that the GATA factor, Cir1, is a major regulator both of the iron uptake functions needed for growth in host tissue and the key virulence factors such as capsule, melanin and growth at 37°C. We are interested in further defining the mechanisms of iron acquisition from inorganic and host-derived iron sources with the goal of understanding the nutritional adaptation of *C. neoformans* to the host environment. In this study, we investigated the roles of the *HAP3* and *HAPX* genes in iron utilization and virulence. As in other fungi, the *C. neoformans* Hap proteins negatively influence the expression of genes encoding respiratory and TCA cycle functions under low-iron conditions. However, we also found that HapX plays both positive and negative roles in the regulation of gene expression, including a positive regulatory role in siderophore transporter expression. In addition, HapX also positively regulated the expression of the *CIR1* transcript. This situation is in contrast to the negative regulation by HapX of genes encoding GATA iron regulatory factors in *Aspergillus nidulans* and *Schizosaccharomyces pombe*. Although both *hapX* and *hap3* mutants were defective in heme utilization in culture, only HapX made a contribution to virulence, and loss of HapX in a strain lacking the high-affinity iron uptake system did not cause further attenuation of disease. Therefore, HapX appears to have a minimal role during infection of mammalian hosts and instead may be an important regulator of environmental iron uptake functions. Overall, these results indicated that *C. neoformans* employs multiple strategies for iron acquisition during infection.

## Author Summary

*Cryptococcus neoformans* causes life-threatening central nervous system infections in immunocompromised people such as AIDS patients. The competition for iron between pathogens such as *C. neoformans* and mammalian hosts is a key aspect of disease outcome. We previously identified and characterized the major iron regulatory protein Cir1 in *C. neoformans*, as well as proteins for the transport of iron-binding molecules (siderophores) and for high-affinity iron uptake. In this study, we examined the roles of additional regulatory proteins (Hap proteins) in the response to low-iron conditions and the use of host iron sources such as heme and transferrin. We discovered that the HapX protein has a conserved regulatory function to repress iron-dependent functions during iron deprivation, as well as a positive regulatory role for the expression of putative siderophore transporters. A *hapX* mutant was defective in the use of heme as an iron source in culture but was only modestly attenuated for virulence in mice. This result suggests that additional mechanisms for iron uptake must be available to support *C. neoformans* proliferation in the host, and that HapX may play an important role in environmental iron acquisition.

## Introduction

The fungus *Cryptococcus neoformans* causes life-threatening illness in immunocompromised individuals. For example, there are an estimated one million cases of cryptococcal meningitis globally per year in AIDS patients, leading to approximately 625,000 deaths [1]. A related species, *Cryptococcus gattii*, has emerged as a primary pathogen of immunocompetent people, as demonstrated by the ongoing occurrence of infections in otherwise healthy people and animals on the West Coast of North America [2]–[4]. These fungi share a set of virulence determinants that include the production of a polysaccharide capsule, the formation of melanin in the cell wall and the ability to grow at 37°C. The capsule makes a major contribution to virulence, and its size is influenced by iron and CO_2_ levels, growth in serum, and host tissue location [5]–[8]. The increase in capsule size in response to iron deprivation is likely to be important in the mammalian environment where iron withholding contributes to host defense [9]. Melanin synthesis is also regulated by iron and additional factors such as glucose [10]–[12].

The influence of iron on capsule size in *C. neoformans* has prompted efforts to define the mechanisms of iron sensing and uptake [13]–[18]. These mechanisms are likely to be important determinants of disease outcome because of the well-documented competition for iron between microbes and mammalian hosts during infection [19]. Initially, the transcriptional response of the fungus to iron deprivation was characterized using serial analysis of gene expression [13]. This transcriptome study identified a set of iron-regulated genes, including the *CAP60* gene that is required for capsule formation, and revealed that defects in an iron-regulated iron permease and an abundant mannoprotein, Cig1, caused growth defects in low-iron medium. A mutation in *CIG1* also altered the regulation of capsule size in response to iron availability. Subsequent work on a siderophore transporter, Sit1, revealed a role in the use of siderophore-bound iron and growth in a low-iron environment, but not in virulence in a mouse model of cryptococcosis [14]. In contrast, loss of the high-affinity iron uptake system comprised of the iron permease Cft1 and the ferroxidase Cfo1 resulted in mutants that were attenuated for virulence but still eventually caused disease [17], [18]. This result suggests that other iron uptake mechanisms are in play during infection, even though Cft1 and Cfo1 are required for growth under low-iron conditions and for utilization of both inorganic iron and iron from transferrin in culture [17], [18]. The interconnections between iron and virulence in *C. neoformans* have recently been reviewed [16].

The mechanisms governing the fungal response to iron deprivation have been best characterized in *Saccharomyces cerevisiae* [20], [21]. In this fungus, iron starvation activates the transcriptional activators Aft1 and Aft2 to induce expression of iron regulon genes including those encoding uptake functions. Induced functions also include the RNA binding proteins Cth1 and Cth2 that mediate the degradation of mRNAs for some iron-dependent proteins [22]. In *Aspergillus nidulans* and *Schizosaccharomyces pombe*, GATA repressors (SreA and Fep1, respectively) and the regulatory subunits of the CCAAT-binding complex (HapX and Php4, respectively) control the expression of iron-dependent genes [23], [24]. For example, the Fep1 repressor in *S. pombe* prevents the expression of iron acquisition functions under iron-replete conditions [23], [25], [26]. In this situation, Fep1 represses the transcription of the *php4+* gene and prevents Php4 from negatively regulating the Php2/3/5 protein complex. This complex activates the transcription of genes for iron storage, the TCA cycle and iron-sulfur cluster biosynthesis when iron is available. These genes are normally repressed by the Php2/3/4/5 complex when insufficient iron is available to support enzyme function; in this condition, Fep1 does not block the transcription of *php4*+. This regulatory scheme has a similar functional outcome as the iron-responsive regulation of mRNA stability by Cth2 in *S. cerevisiae* [22]. Specifically, the transcription of genes encoding iron-requiring proteins is repressed upon iron deprivation thus allowing the cells to spare iron for essential functions. In *A. nidulans*, SreA plays a parallel role to that of Fep1 and HapX functions in a similar manner to Php4 [24].

In *C. neoformans*, the GATA factor Cir1 is a major regulator of both the transcriptional response to iron and the expression of virulence factors [15]. Cir1 shows sequence similarity to the regulators Fep1 and SreA but lacks one of the two zinc finger motifs found in these factors. Mutants defective in *CIR1* are transcriptionally unresponsive to iron deprivation and exhibit iron-related phenotypes. Transcriptional profiling of wild type and *cir1Δ* cells revealed that Cir1 plays both positive and negative regulatory roles for the majority of genes encoding iron uptake functions as well as genes for a large number of other functions (signal transduction, transcription, sterol biosynthesis, DNA replication and cell wall biosynthesis). For example, Cir1 positively regulates the expression of the genes *CFT1* and *CFO1* that encode the high affinity iron uptake system, and negatively regulates genes encoding candidates for intracellular iron transport (*CFT2*, *CFO2*) and the *LAC1* gene encoding laccase [15], [17], [18]. Interestingly, loss of Cir1 also altered the expression of all of the major virulence factors including loss of capsule formation, enhanced melanin deposition and poor growth at 37°C. Given these changes, it was not surprising a *cir1Δ* mutant was completely avirulent in a mouse model of cryptococcosis. These findings demonstrate that the iron regulatory network is a critical aspect of pathogenesis in *C. neoformans*.

In addition to Cir1, transcription factors with demonstrated or potential roles in iron regulation include the *C. neoformans* ortholog of the pH–responsive factor *RIM101* [27], [28], a *HAPX* ortholog identified by Hortschansky et al. [24] and the *HAP3* and *HAP5* components of the CCAAT-binding complex that interacts with HapX in other fungi [24], [29], [30]. A number of other transcription regulators have been found to regulate subsets of genes encoding iron uptake functions in *C. neoformans* including Tup1, Nrg1 and Sre1 [31]–[34]. In this study, we examined the roles of the *HAP* genes in iron-related phenotypes, in the transcriptional response to different iron sources, and in virulence. We also examined the regulatory connections between Cir1, HapX and Rim101 at the transcriptional level. We found that HapX shares conserved regulatory functions with other fungi with regard to repression of iron-dependent functions during iron deprivation. However, we also discovered that HapX has a positive regulatory role for *CIR1* and for a subset of genes that encode putative siderophore transporters. A mutant defective in HapX did not show virulence-related phenotypes in culture, but was modestly attenuated for virulence in mice. In contrast, Hap3, as a representative of the CCAAT-binding complex, showed virulence-related phenotypes in culture, but did not contribute to virulence. Surprisingly, deletion of *HAP3* or *HAPX* in the background of a *cfo1Δ* mutant did not cause further attenuation of virulence relative to the *cfo1Δ* mutant alone. This result suggests that additional mechanisms for iron acquisition must be available to support *C. neoformans* proliferation in the host environment.

## Results

### Defects in the *HAP3*, *HAP5* and *HAPX* genes attenuate growth on hemin

The *HAP* genes were initially examined with mutants in the deletion collection constructed by Liu et al. [28] to investigate their roles in the regulation of iron acquisition. The deletion collection contained mutants for two genes related to *HAP3* in *S. cerevisiae* (designated *HAP3-1* and *HAP3-2*), mutants for *HAP5* and *HAPX*, as well as a strain with a defect in a putative *HAP1* gene. The growth of these strains on solid media containing different iron sources initially revealed that three mutants (*hap3-1Δ*, *hap5Δ* and *hapXΔ*) had poor growth on hemin (the ferric iron version of heme) (Figure S1). We subsequently reconstructed the mutants for the *HAP3*, *HAP5* and *HAPX* genes singly and in combination with the *cfo1Δ* (ferroxidase) mutation in the background of the serotype A strain H99. This strain has been used to characterize iron uptake because of its high virulence in a mouse model of cryptococcosis [14], [15], [17], [18].

**Figure 1:**
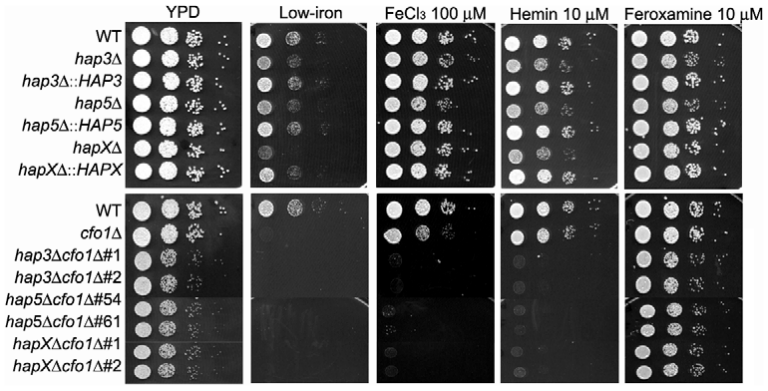
The *hap3Δ, hap5Δ* and *hapXΔ* mutants grow poorly on low-iron media supplemented with hemin. Ten-fold serial dilutions of cells (starting at 10^4^ cells) were spotted onto solid YPD medium, solid YNB medium (control), low-iron medium (YNB+100 µM BPS), low-iron medium supplemented with 100 µM FeCl_3_, 10 µM Hemin or 10 µM Feroxamine. The bottom panel shows a comparison of the growth of the ferroxidase mutant *cfo1Δ* with double mutants lacking the *CFO1* gene and the *HAP3*, *HAP5* or *HAPX* gene. For the double mutants, two independent strains of each mutant were tested as indicated. The plates were incubated at 30°C for two days.

As shown in Figure 1, the wild type (WT) and the reconstructed mutant strains all had weak growth on low-iron medium, with especially poor growth observed for the *hapXΔ*, *cfo1Δ* and double mutants. Growth was restored for all of the strains upon addition of the siderophore feroxamine; however, ferric chloride allowed growth of all of the single mutants but not the double mutants lacking the *HAP* genes and *CFO1*. The *hap3Δ*, *hap5Δ* and *hapXΔ* mutants each showed weaker growth on medium with hemin as the sole iron source, and complementation with the corresponding genes restored growth to the WT level. Similarly, the double mutants all failed to grow on hemin. The poor growth of the mutants with hemin was further confirmed with assays in liquid media (Figure S2). The sequence alignments, deletion constructs and confirmation of the mutant genotypes are shown in Figures S3 and S4. Taken together, these results indicated that Hap3, Hap5 and HapX all contribute to iron utilization from hemin (e.g., by regulating uptake and/or processing functions); this finding prompted a further examination of the impact of *hap* mutations on iron-related transcription and virulence.

### HapX plays a greater role than Cir1 and Hap3 in the transcriptional response to inorganic and mammalian iron sources

The influence of the *hapXΔ*, *hap3Δ* and *cir1Δ* mutations on transcription was examined by microarray analysis of RNA from cells incubated in low-iron media alone or in the presence of ferric chloride, hemin or transferrin. As described in the Materials and Methods, three biological replicates of iron-starved cells were prepared for each strain and the cells were grown for 6 h in each iron condition prior to RNA extraction. The time point of 6 h is just before the initiation of growth in each of the iron sources, although the mutants failed to grow in low-iron medium and medium with transferrin (Figure S2). For the microarray experiments, cDNA synthesized from the total RNA was hybridized to oligonucleotide arrays initially designed for genes in the serotype D strain JEC21 and updated to include genes for the strain H99 employed in our experiments (see Materials and Methods). In total, the hybridization signals for 6165 oligonucleotides that matched H99 genes (Table S1) were evaluated.

**Figure 2:**
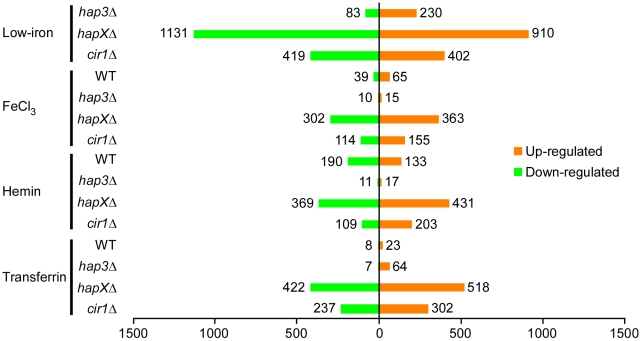
Overview of differentially-regulated genes in response to different iron sources. A histogram shows the number of genes whose expression changed by at least two-fold with statistical significance (*q*<0.05). The numbers represent differentially expressed genes in the mutants versus WT in response to different iron sources and also in the WT strain grown under iron-replete versus iron-limited conditions.

Initially, we examined the global changes in transcription for each mutant in comparison with the WT strain in each iron source (Figure 2). This analysis revealed that the *hapXΔ* mutation had the greatest influence, with 1131 genes down-regulated and 910 up-regulated upon comparison with WT under low-iron conditions. By contrast, Hap3 made a much smaller contribution with 83 genes down-regulated and 230 up-regulated under the same conditions. The *cir1Δ* mutant had an intermediate influence with 419 and 402 genes down- and up-regulated, respectively. The latter numbers are consistent with our previous analysis of the influence of Cir1 on transcription in a serotype D strain of *C. neoformans* [15]. Overall, the same global pattern of influence was observed with the cells grown with the three iron sources, although the numbers of genes influenced by the mutations were lower by 30 to 50% compared with the differential expression seen under low-iron conditions. Also, these results indicated that HapX plays a major role in the regulation of gene expression under low-iron conditions. Venn diagrams displaying the overlapping numbers of genes for each mutation and each iron condition are presented in Figure S5. The complete lists of differentially expressed genes that correspond to the numbers in the Venn diagrams are presented in Tables S2, S3, S4, S5.

### HapX negatively regulates functions for electron transport and positively regulates genes encoding siderophore transporters in response to low-iron conditions

**Figure 3:**
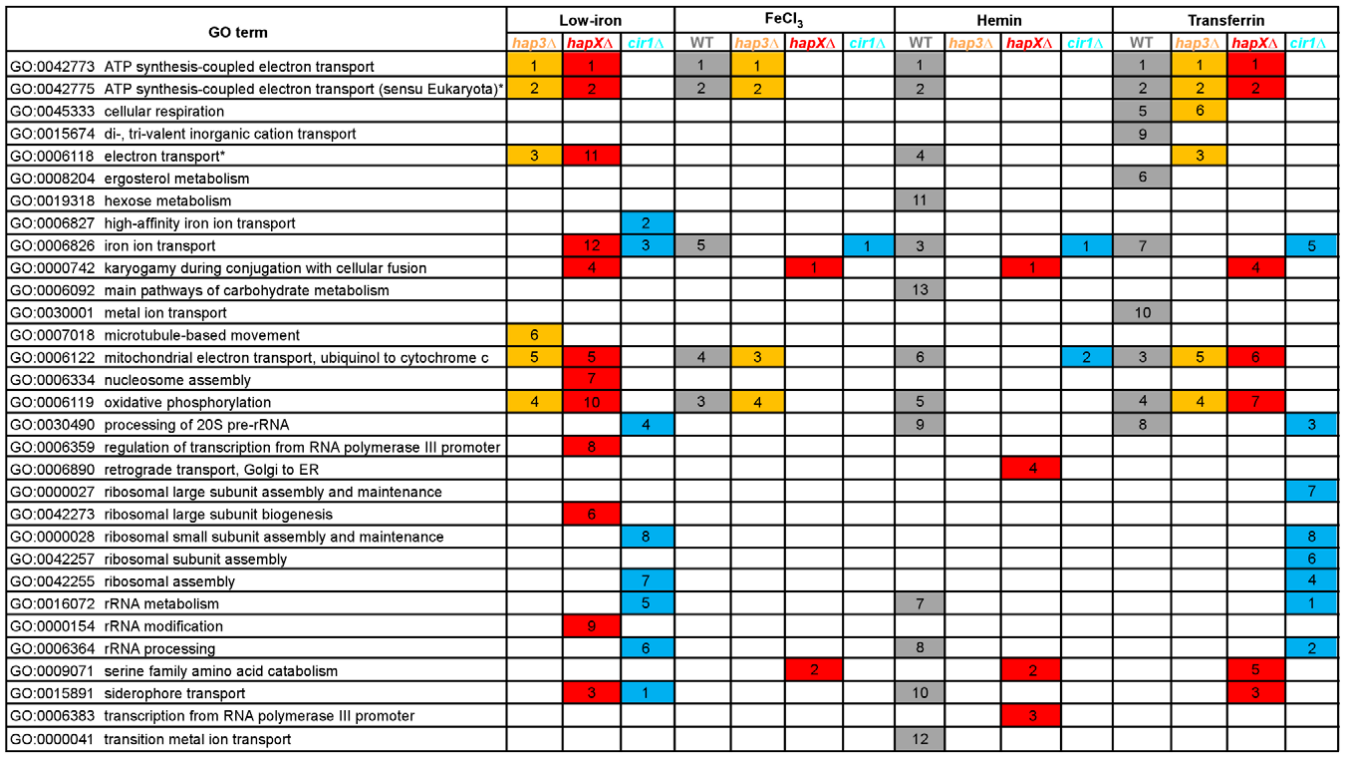
Enriched GO terms for differentially-regulated genes in the mutants and WT strain in response to different iron sources. The figure shows the GO terms identified by Gene Score Re-sampling with the microarray data mining tool ermineJ for differentially expressed genes in the mutants versus WT in response to different iron sources. The terms are also shown for WT in iron-replete versus iron-limited conditions. For each comparison, GO terms with statistical significance (*p*<0.05) are indicated by colored boxes. The numbers within the colored boxes represent the GO term rankings within each comparison. Two GO terms (indicated by asterisk) from the ermineJ annotation file are updated on the GO website (http://www.geneontology.org/): GO:0042775 is described as mitochondrial ATP synthesis coupled electron transport and GO:0006118 has been made obsolete and replaced by GO:0055114 oxidation reduction.

We next examined the Gene Ontology (GO) terms in the microarray data to identify the enriched functions among the differentially expressed genes in each mutant and comparison. As described in our previous analysis of Cir1 [15], we employed the data mining tool ermineJ to identify the GO terms listed in Figure 3 [35]. Supplemental Tables S6 and S7 list the GO terms and the genes in each GO term, along with their *p* values, respectively. The top GO terms for the *hapXΔ* mutant versus WT under low-iron conditions were ATP synthesis-coupled electron transport (two categories, (Figure 3 and Table 1) followed by siderophore transport, karyogamy during conjugation with cellular fusion (mainly tubulin genes), and mitochondrial electron transport. Further analysis of the GO terms for mutant versus WT indicated that HapX does not show differentially expressed genes in inorganic iron- or hemin-replete conditions (except in specific categories other than direct iron uptake functions, such as karyogamy, serine family amino acid catabolism, retrograde transport and RNA polymerase III transcription). Thus, it appeared that HapX primarily influences differential gene expression under low-iron conditions and to a lesser extent with transferrin. The lower response to transferrin as an iron source may reflect the timing of the analysis in that the cells may take longer to respond to this iron source compared to ferric chloride or hemin. The influence of HapX under low-iron conditions served to focus attention on a specific subset of iron regulon genes related to siderophore transport (and iron ion transport to a lesser extent) (Table 2). Interestingly, genes in the category “high-affinity iron ion transport” were not influenced by loss of HapX. This result suggests that, in contrast to the more global influence of Cir1 (see below), the influence of HapX on the expression of iron uptake functions may be restricted to the subset of genes needed for siderophore uptake. The differential transcript levels for a subset of the genes in Tables 1 and 2 were confirmed by quantitative RT-PCR (data not shown).

**Table 1:** HapX and Hap3 negatively regulate the expression of genes encoding iron-dependent functions.

Microarray Oligo ID	H99 gene ID^a^	Gene annotation	Fold change (Low-iron)^b^		
			*hap3Δ* vs WT	*hapXΔ* vs WT	*cir1Δ* vs WT
163.m06475	CNAG_01287	NADH-ubiquinone oxidoreductase 51 kDa subunit	16.09	8.18	−1.20*
163.m06504	CNAG_01323	ubiquinol-cytochrome-c reductase	5.68	5.24	−1.00*
167.m03416	CNAG_02266	NADH ubiqionone oxidoreductase chain B	1.24*	1.41*	−1.67*
167.m03373	CNAG_02315	ubiquinol-cytochrome c reductase iron-sulfur¥	5.72	11.98	−1.57
177.m03188	CNAG_03226	succinate dehydrogenase iron-sulfur subunit¥	47.64	48.03	−1.17*
186.m03551	CNAG_03629	NADH-ubiquinone oxidoreductase	7.63	6.78	−2.08*
162.m02855	CNAG_04189	succinate dehydrogenase flavoprotein subunit	21.10	21.50	−1.04*
184.m04775	CNAG_05179	ubiquinol-cytochrome C reductase complex core	4.13	2.46	−1.54*
184.m04608	CNAG_05631	NADH-ubiquinone oxidoreductase	12.32	17.17	−1.53*
CNAG_05633^c^	CNAG_05633	hypothetical protein	3.93	5.13	−2.11
180.m02996	CNAG_05909	electron transporter	5.08	4.38	−2.35
CNAG_06663^d^	CNAG_06663	conserved hypothetical protein	7.86	11.35	−1.14*
180.m00153	CNAG_07177	NADH-ubiquinone oxidoreductase kDa subunit	1.55*	1.71*	−1.63*
181.m08761	CNAG_07356	succinate dehydrogenase¥	30.61	33.41	1.66*

a All the genes, except CNAG_03629, are associated with both the GO terms ATP synthesis-coupled electron transport (GO:0042773) and ATP synthesis-coupled electron transport (sensu Eukaryota) (GO:0042775). CNAG_03629 is only associated with GO:0042773. On the GO website, the GO term for GO:0042775 is described as mitochondrial ATP synthesis-coupled electron transport.

b Values statistically significant with *p*<0.05, except those marked with *.

c abbreviation of CNAG_05633 hypothetical protein (526 nt).

d abbreviation of CNAG_06663 conserved hypothetical protein (917 nt).

*Values statistically not significant.

**¥:** Validated by quantitative real-time RT-PCR.

**Table 2:** HapX positively regulates the expression of genes encoding iron acquisition functions.

GO term	Microarray Oligo ID	H99 gene ID	Gene annotation	Fold change (Low-iron)^a^		
				*hap3Δ* vs WT	*hapXΔ* vs WT	*cir1Δ* vs WT
Siderophore transport (GO:0015891)	181.m08534	CNAG_00815	siderochrome-iron uptake transporter (Sit1)¥	−2.09	−4.09	−10.94
	167.m03592	CNAG_02083	siderochrome-iron transporter¥	1.06*	−3.81	−14.65
	186.m03450	CNAG_06761	siderophore-iron transporter Str1¥	−3.55	−19.71	−2.55*
	181.m07976	CNAG_07334	ferric reductase transmembrane component	1.78*	1.13*	5.79
	181.m08575	CNAG_07387	siderophore-iron transporter Str3¥	1.02*	−2.01*	−1.02*
	CNAT_07061^b^	CNAG_07519	conserved hypothetical protein¥	−1.71*	−20.20	−5.22
	177.m02867	CNAG_07751	siderophore iron transporter mirB¥	−2.98	−31.82	1.03*
High-affinity iron ion transport (GO:0006827)	CNAG_02959^c^	CNAG_02959	high-affinity iron permease CaFTR1 (Cft2)¥	1.05*	2.93	7.02
	177.m02973	CNAG_03465	laccase (Lac1)¥	1.40*	−1.41*	45.70
	186.m03615	CNAG_03694	iron ion transporter	−1.82	−2.40	−2.81
	162.m02793	CNAG_04293	vacuolar protein sorting 41¥	1.95*	3.78	4.12
	164.m02206	CNAG_06241	acidic laccase (Cfo1)¥	−1.44	−1.03*	−2.81
	164.m02187	CNAG_06242	high-affinity iron permease CaFTR1 (Cft1)¥	−1.13*	−1.38*	−1.88*
	185.m02701	CNAG_07865	ferro-O2-oxidoreductase	−1.94*	−5.06	1.14*

a Values statistically significant with *p*<0.05, except those marked with *.

b abbreviation of CNAT_07061_CNAG_07061_cneo.

c abbreviation of CNAG_02959 high-affinity iron permease CaFTR1 (2178 nt).

*Values statistically not significant.

**¥:** Validated by quantitative real-time RT-PCR.

### Hap3 negatively regulates genes for electron transport but has a minimal influence on the expression of iron uptake functions

The top ranked GO terms for the *hap3Δ* mutant versus WT under low-iron conditions were ATP synthesis-coupled electron transport (two categories) followed by electron transport, oxidative phosphorylation and mitochondrial electron transport (Figure 3). Several of these GO terms overlapped those observed with the *hapXΔ* mutation under low-iron conditions, and this suggests that these factors may function together in the regulation of specific categories of genes. For example, both Hap3 and HapX negatively influenced the expression of electron transport functions (Table 1). It is therefore possible that HapX works with Hap3 and the other CCAAT-binding complex proteins to repress iron-dependent functions under low-iron conditions, as seen in other organisms [24], [25]. Tables S2, S3, S4, S5 provide the lists of the specific genes that are differentially regulated in each of the iron conditions. The overlap in GO terms for the *hapXΔ* and *hap3Δ* mutants was not seen for iron-replete conditions with inorganic iron or hemin, but the pattern was observed with transferrin (Figure 3). As mentioned above, this result may reflect experimental conditions in that this iron source may not be used as readily or at the same rate as other iron sources. Alternatively, there may be a separate regulatory program for transferrin utilization. Loss of Hap3 had a minimal influence on the transcription of the genes for iron transport (Table 2), thus highlighting a regulatory difference compared to HapX. As mentioned above, the GO terms for iron transport influenced by HapX but not Hap3 represent positive regulatory influences of HapX primarily on siderophore transporters (Table 2). It is also interesting to note that loss of Hap3 resulted in only 28 differentially expressed genes upon comparison with WT grown on hemin (Figure 2). In fact, no enriched GO terms were found for the *hap3Δ* mutant in this comparison (Figure 3). Overall, it appears that Hap3 plays a less prominent role than HapX in the transcriptional response of *C. neoformans* to different iron sources, and that the majority of the Hap3 influence is on electron transport functions, as is found in other fungi [24], [25].

### Cir1 and HapX regulate overlapping subsets of genes encoding iron transport functions

The top GO terms for the *cir1Δ* mutant versus WT under low-iron conditions were siderophore transport, high-affinity iron ion transport, iron ion transport, processing of 20S pre-RNA, and rRNA metabolism (Figure 3). The inclusion of the *cir1Δ* mutant in the analysis allowed a comparison of overlapping regulatory activities for the Hap transcription factors and Cir1. We were particularly keen to determine whether Cir1 and HapX co-regulated specific genes for iron acquisition functions. As described above, HapX has the negative regulatory function to control iron-dependent functions (e.g., electron transport) during iron deprivation. However, it also has a positive activity for the expression of iron regulon genes for siderophore transport. The examination of the GO terms for differentially expressed genes of HapX and Cir1 revealed that the only shared categories were iron ion transport and siderophore transport under low-iron conditions. No other shared categories were identified under any of the iron-replete conditions. In addition, there was no overlap in the GO terms regulated by Cir1 and Hap3. Thus, it appears that HapX and Cir1 may partner in the regulation of genes for iron acquisition related to siderophore transport (Table 2). For example, both HapX and Cir1 positively regulated the transcript level of the *SIT1* gene that was previously shown to function in siderophore transport [14]. In addition, Cir1 regulates genes of relevance for virulence that are not regulated by HapX or Hap3 and a clear example is the *LAC1* gene encoding the laccase for melanin formation.

### HapX positively regulates *CIR1* and *RIM101* transcript levels

To further explore the relationship between *HAPX* and *CIR1*, the microarray data were examined for the expression patterns of these genes in each mutant background. In addition, we included *RIM101*, a gene encoding a pH-responsive transcription factor, because O'Meara et al. [27] recently reported an influence of Rim101 on the expression of iron uptake genes. Several of these genes were included in the GO terms that we observed to be regulated by Cir1 (high-affinity iron ion transport, iron ion transport and siderophore transport) and HapX (iron ion transport and siderophore transport). For example, Rim101 positively regulated the transcript levels of several siderophore transporter genes including *SIT1*, as well as the *CFT1* gene encoding the iron permease that is required for full virulence [14], [17]. We initially focused on the low-iron condition for this analysis and found that Cir1 had a minor influence on the transcript level for *HAPX* in this situation. This observation agreed with previous microarray analysis of the *cir1Δ* mutant in a serotype D strain [15]. In contrast, Cir1 had a positive influence on the *RIM101* transcript with a 6.4-fold higher level in the WT strain compared with the *cir1Δ* mutant. We also found that HapX positively influenced levels of both the *CIR1* transcript (3.5-fold) and the *RIM101* transcript (2.0-fold) under low-iron conditions. Under iron-replete conditions, Cir1 positively regulated *RIM101* (4.0-fold) but did not significantly influence the expression of *HAPX*. Under this condition, HapX showed no regulation of *RIM101* and only a small positive influence on *CIR1* transcript levels (1.3-fold), that was not statistically significant.

**Figure 4:**
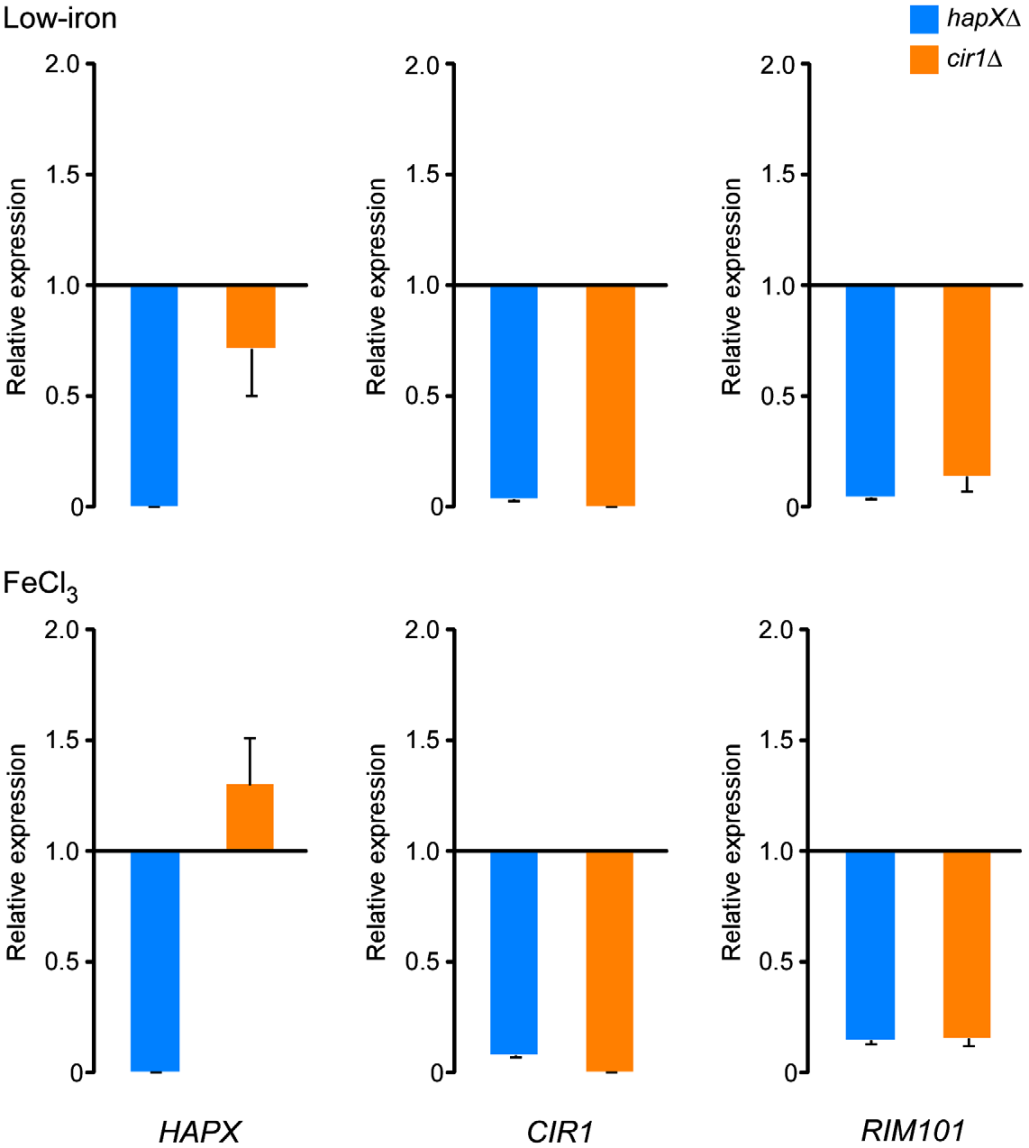
HapX positively regulates the levels of the *CIR1* and *RIM101* transcripts. Quantification of *HAPX*, *CIR1* and *RIM101* transcripts by quantitative RT-PCR in WT, *hapXΔ* and *cir1Δ* strains under low-iron (top) and iron-replete (bottom) conditions. The data were normalized using 18S rRNA as an internal control and are presented as relative expression in comparison to the WT value set at 1. The data are from three biological and two technical replicates, and the bars represent standard deviations.

Overall, the microarray data therefore indicated interconnected levels of regulation for the three transcription factors. To confirm the relationships between the transcription factors, we employed quantitative RT-PCR with RNA prepared from the *cir1Δ* and *hapXΔ* mutants grown under low-iron and iron-replete conditions. As shown in Figure 4, the quantitative RT-PCR analysis supports the conclusion that HapX has a positive regulatory influence on the transcript levels of both *CIR1* and *RIM101*. We also noticed that a larger influence of HapX on the *CIR1* transcript was found under the iron-replete condition by quantitative RT-PCR. This may reflect the greater sensitivity of this method relative to microarray analysis. We should also note that O'Meara et al. [27] performed a microarray comparison of the *rim101* mutant with the WT parental strain and found that Rim101 positively influences the expression of *HAPX* (4.8-fold).

### Hap3 and Hap5 influence capsule size, growth at host temperature and utilization of non-fermentable carbon sources

**Figure 5:**
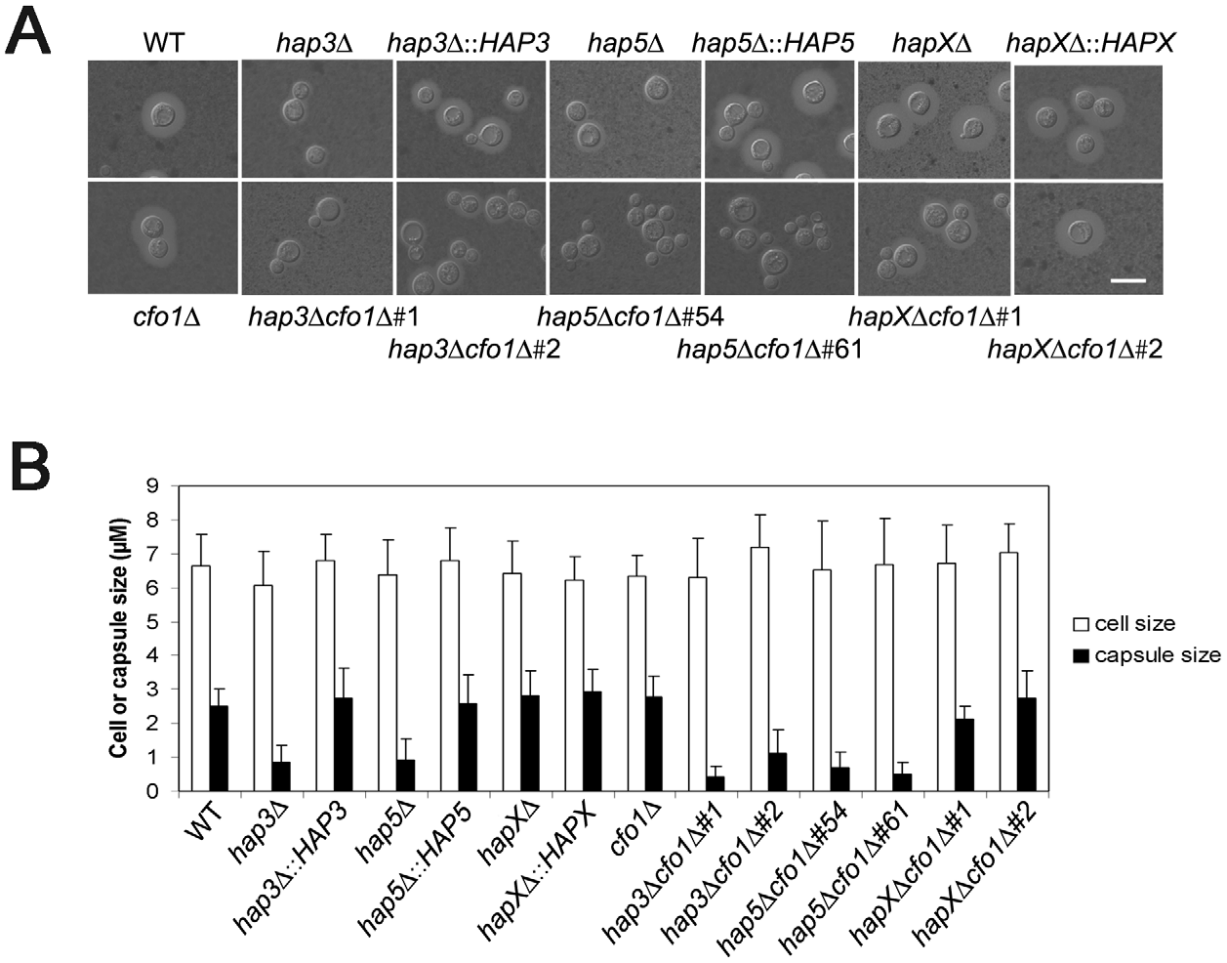
The *hap3Δ* and *hap5Δ* mutants have a smaller capsule than WT or *hapXΔ* cells. **A**. Cells were cultured in low-iron medium (LIM) at 30°C for 48 h. Capsule formation was assessed by India ink staining for the WT strain, the *hap3Δ*, *hap5Δ*, *hapXΔ*, *cfo1Δ*, *hap3Δ cfo1Δ*, *hap5Δ cfo1Δ*, and *hapXΔ cfo1Δ* mutants, as well as the reconstituted strains. Bar = 10 µ. **B**. Fifty cells from each strain were measured to determine cell diameter and capsule radius. The capsule sizes of *hap3Δ*, *hap5Δ*, *hap3Δ cfo1Δ* (both #1 and #2) and *hap5Δ cfo1Δ* (both #54 and #61) cells were statistically different (t-test, *p*<0.001) from the sizes for cells of the WT strain, the *hapXΔ cfo1Δ* mutants and the reconstituted strains. Each bar represents the average of 50 measurements with standard deviations.

We next examined the *hap3Δ*, *hap5Δ*, and *hapXΔ* mutants, and the double mutants lacking the *HAP* genes and *CFO1*, for expression of the three major virulence factors: capsule, melanin, and growth at 37°C. Initially, we found that the *hap3Δ* and *hap5Δ* mutants (and the double mutants for these genes and *cfo1Δ*) produced a smaller capsule than the WT strain or the *hapX* mutant (Figure 5). Complementation with the *HAP3* and *HAP5* genes restored normal capsule size. We also found that all of the mutants displayed WT levels of melanin formation when tested on medium containing the substrate L-DOPA (data not shown). This result was consistent with the lack of an influence of the *hap3Δ* or *hapXΔ* mutations on the transcript levels for the laccase gene *LAC1* (gene ID: CNAG_03465).

**Figure 6:**
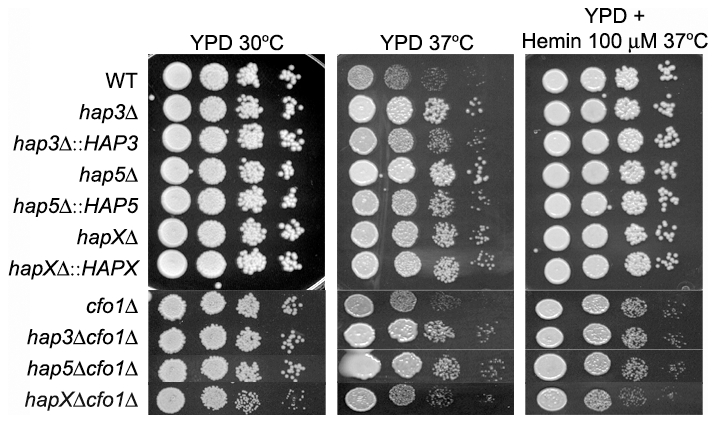
The *hap3Δ* and *hap5Δ* mutants exhibit enhanced growth at host temperature. Ten-fold serial dilutions of WT and mutant cells (starting at 10^4^ cells) were spotted onto solid YPD medium with no addition or with 100 µM Hemin and incubated at the indicated temperatures for two days.

With regard to growth at host temperature, we made the surprising observation that the *hap3Δ* and *hap5Δ* mutants (with or without the *cfo1Δ* mutation) showed more robust growth at 37°C than the WT or the *hapXΔ* mutant (Figure 6). Interestingly, addition of hemin enhanced the growth of the other strains suggesting an unexpected interplay between this iron source and temperature. We previously observed enhanced growth in response to hemin for the *C. neoformans cfo1Δ* mutant (defective in the ferroxidase for high-affinity iron uptake) upon exposure to the antifungal drug fluconazole [18]. Presumably, this phenomenon is due to the requirement of enzymes in the ergosterol biosynthesis pathway for heme and connections between iron availability and heme biosynthesis. As part of our microarray analysis, we found that Hap3 and HapX did regulate the expression of the genes encoding ergosterol and heme biosynthetic functions under low-iron conditions (Tables S8 and S9).

**Figure 7:**
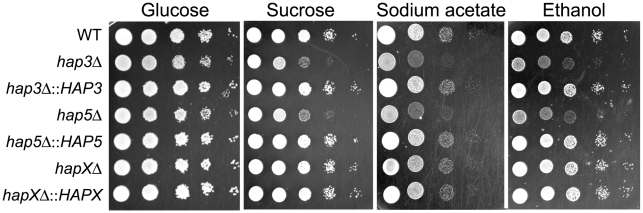
The *hap3Δ* and *hap5Δ* mutants exhibit poor growth on acetate, ethanol and sucrose. Ten-fold serial dilutions of the WT, *hap3Δ*, *hap5Δ* and *hapXΔ* mutants and the reconstituted strains (starting at 10^4^ cells) were spotted on YNB supplemented with the indicated carbon sources (all at 2%). Plates were incubated at 30°C for two (on glucose and sucrose) or three (on acetate and ethanol) days.

We also examined the carbon source requirements for the *hap* mutants because it is known that the *HAP* genes in other fungi regulate growth on non-fermentable carbon sources [29], [30]. The *C. neoformans* mutants were tested for growth on glucose, sucrose, acetate and ethanol, and it was found that the *hap3Δ* and *hap5Δ* mutants showed weaker growth on the latter three carbon sources (Figure 7). Unexpectedly, the *hapXΔ* mutant did not show these growth phenotypes even though the transcriptional profiling suggested that HapX and Hap3 both participate in the regulation of genes needed for respiratory growth (Figure 3). It is possible that the growth differences between the *hap3Δ* and *hapXΔ* mutants reflect differential influences on the expression of respiratory functions under the iron-replete conditions in the media. The addition of hemin did not rescue the growth defects of the *hap3Δ* and *hap5Δ* mutants suggesting that lack of this iron source is not responsible for the observed phenotypes on acetate and ethanol (data not shown). Overall, these analyses provided several examples of distinct phenotypes for the *hapXΔ* mutant relative to the *hap3Δ* and *hap5Δ* mutants. In a further example, we noted that the *hap3Δ* and *hap5Δ* mutants were more sensitive to growth in the presence of 0.01% SDS, compared with the *hapXΔ* mutant and the WT strain (data not shown). As described below, the distinct behavior of the *hapXΔ* mutant extends to virulence.

### Loss of HapX results in a modest virulence defect

**Figure 8:**
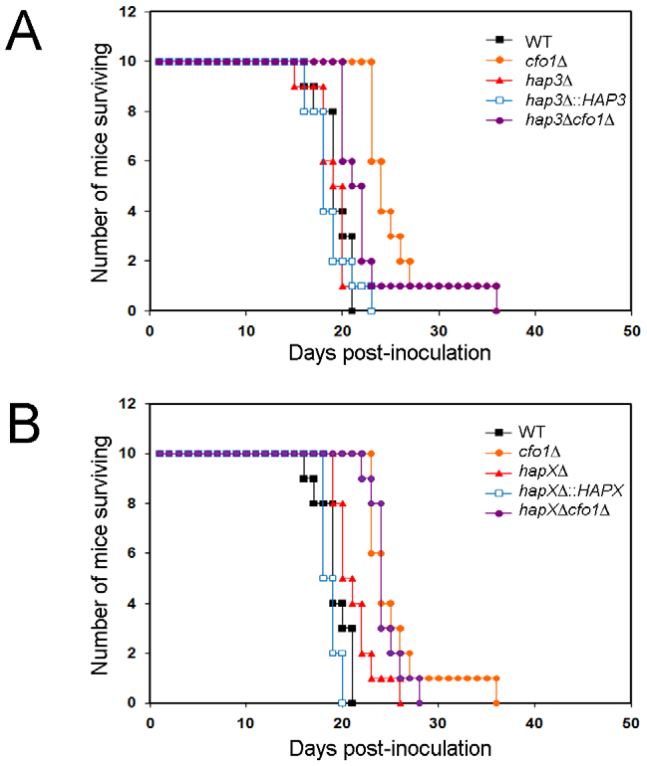
The *hapXΔ* mutant is modestly attenuated for virulence in a mouse inhalation model of cryptococcosis. A. Ten female BALB/c mice were infected intranasally with 5×10^4^ cells of each of the strains indicated (H99 (WT), *cfo1Δ*, *hap3Δ*, *hap3Δ::HAP3*, and *hap3Δ cfo1Δ*) and the survival of the mice was monitored twice per day. Assessment of the *p*-values for survival did not reveal significant differences between WT-, *hap3Δ::HAP3*- and *hap3Δ-*infected mice, or for comparisons of survival for the *cfo1Δ* mutant versus the *hap3Δ cfo1Δ* double mutant. The WT and *cfo1Δ* strains show differences in virulence as previously reported [18]. B. Parallel virulence tests were performed for the *hapXΔ*, *hapXΔ::HAPX*, *and hapXΔ cfo1Δ* strains. Note that the same data for the WT (H99) strain and the *cfo1Δ* mutants are presented in both figures to allow comparisons with the *hap* mutants. Assessments of survival revealed significant differences between WT or the *hapXΔ::HAPX* strain and *hapXΔ* (*p*<0.05); however, no significant difference was found upon comparison of the survival for the *cfo1Δ* mutant versus the *hapXΔ cfo1Δ* double mutant.

The small capsule phenotype of the *hap3Δ* mutant, and the growth defects of the *hap3Δ* and *hapXΔ* mutants on hemin as the sole iron source (particularly when combined with the *cfo1Δ* mutation), prompted an investigation of the virulence of these mutants in a mouse inhalation model of cryptococcosis. We therefore inoculated mice with the WT strain, the *cfo1Δ* mutant and the *hap3Δ* or *hapXΔ* mutants. We also included the complemented mutants and the *hap3Δ cfo1Δ* and *hapXΔ cfo1Δ* double mutants. The rationale for including *cfo1Δ* in the analysis came from previous observations that loss of the Cfo1 ferroxidase or the iron permease Cft1 resulted in attenuated virulence [17], [18]. The Cfo1/Cft1 high-affinity iron uptake system is needed for the use of transferrin but not hemin in culture [17], [18]. We reasoned that loss of Cfo1 and the Hap proteins might interfere with the utilization of both transferrin and hemin, and further attenuate virulence beyond that observed for the *cfo1Δ* mutant alone. As seen in Figure 8A, deletion of *HAP3* did not result in a virulence defect despite the growth defect on hemin (Figure 1) and the small capsule size observed in culture (Figure 5). Also, the double mutant, *hap3Δ cfo1Δ*, showed a delayed virulence phenotype similar to that of the *cfo1Δ* single mutant thus indicating no additive influence.

In contrast to the situation with the *hap3Δ* mutant, the infection of mice with the *hapXΔ* mutant revealed a modest attenuation of virulence manifested by a prolonged survival period of 3–5 days compared with infection with the WT strain (*p* = 0.033; Figure 8B). In this case, reintroduction of the *HAPX* gene resulted in a WT level of virulence compared with the *hapXΔ* mutant (*p* = 0.001). As with the *hap3Δ cfo1Δ* double mutant, the *hapXΔ cfo1Δ* double mutant also showed attenuated virulence in comparison with the WT strain but not when compared with the *cfo1Δ* single mutant. The results for the double mutants indicated that the inability to use hemin as a sole iron source *in vitro* did not translate into a defect in iron acquisition in mice that would attenuate virulence. This result suggests that one or more additional iron acquisition systems, beyond Cfo1 and those influenced by Hap3 and HapX, must function during infection.

### Evidence for an additional iron uptake system under acidic conditions

**Figure 9:**
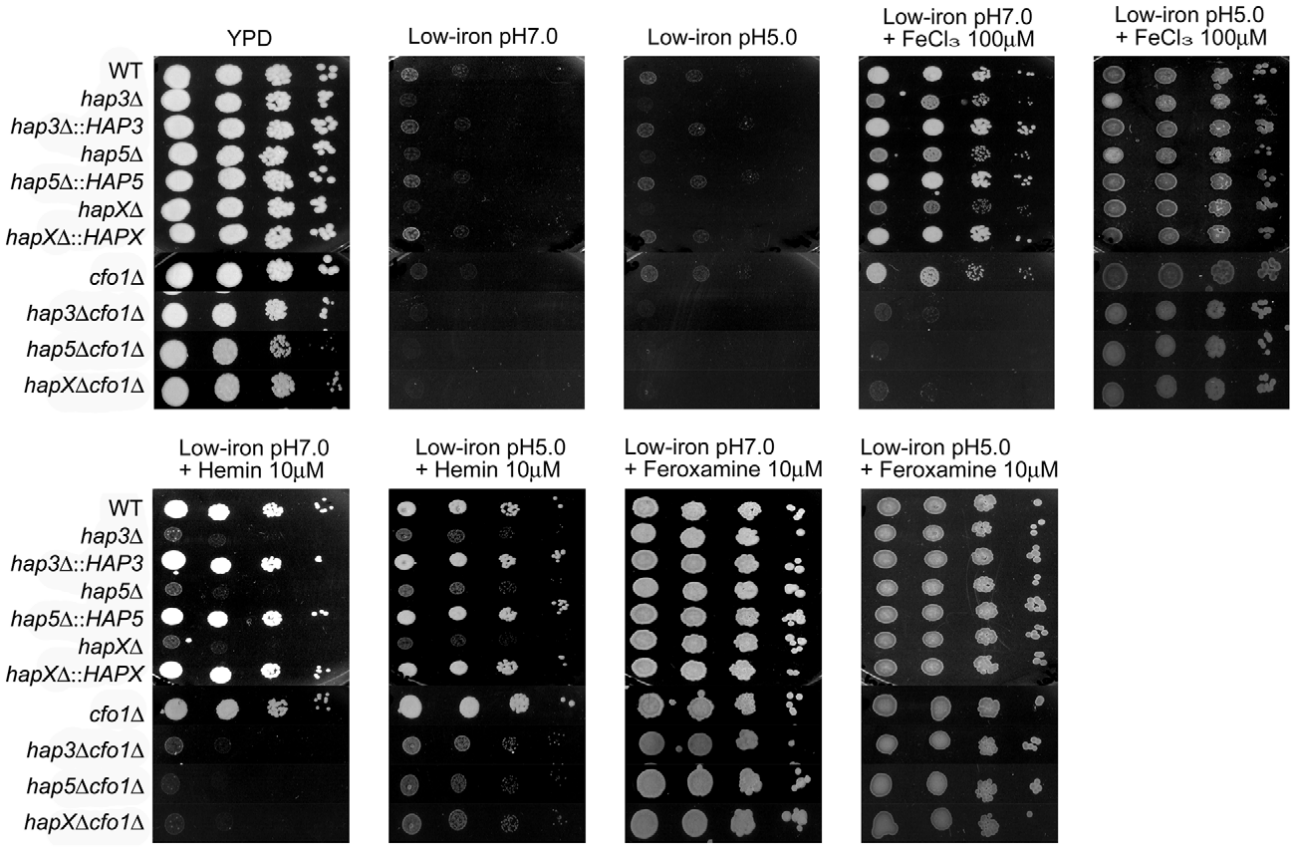
Acidic pH rescues the growth of double mutants on ferric chloride and hemin. Ten-fold serial dilutions of cells (starting at 10^4^ cells) were spotted onto solid YPD medium, low-iron medium (YNB+100 µM BPS) at either pH 5.0 or 7.0, and low-iron medium supplemented with 100 µM FeCl_3_, 10 µM Hemin or 10 µM Feroxamine at each of the two pH conditions. The plates were incubated at 30°C for two days.

Possibilities for additional iron uptake mechanisms include low-affinity or ferrous iron-specific systems that may respond to iron mobilized by reduced pH during infection. A reduction in pH increases iron availability and acidic metabolites such as acetate are known to accumulate during cryptococcal infection [36]. In addition, a low-affinity uptake system for inorganic iron was previously identified in *C. neoformans* by physiological experiments, and intracellular residence of fungal cells in the acidic phagolysosomal compartment may also influence iron availability [37]. We tested the possibility of an additional uptake function by reexamining the growth of the *hap* mutants alone or in combination with the *cfo1Δ* mutation on medium at reduced pH (Figure 9). The plate assays revealed that a reduction in pH to 5.0 rescued the growth of the double mutants on both ferric chloride and hemin in support of the idea that iron availability is enhanced under acidic conditions to allow uptake via an alternative mechanism(s). The results also suggest that the cells require Hap functions to utilize both iron sources in the situation where the loss of Cfo1 activity causes a defect in the high-affinity, reductive iron uptake pathway.

## Discussion

### The roles of Hap3, 5 and X in hemin utilization and virulence

Our goal is to understand the mechanisms of iron sensing and acquisition employed by *C. neoformans* in the environment and in iron-deprived niches of mammalian hosts. This information may enable new therapeutic approaches to reduce the substantial global burden of cryptococcosis [1]. Previously, we demonstrated that the GATA factor Cir1 is a major regulator of both iron acquisition and virulence in *C. neoformans* [15]. In this study, we examined the roles of additional regulatory proteins of the CCAAT-binding complex (Hap3 and Hap5) and HapX to further investigate the response to iron deprivation. These proteins are important in the response to low-iron in *S. pombe* and *A. nidulans*, and we hypothesized that they would play a similar role and contribute to virulence in *C. neoformans* [23]–[25]. Indeed, we found that the *hap3Δ*, *hap5Δ* and *hapXΔ* mutants had defects in hemin utilization and, when combined with a mutation in the ferroxidase gene *CFO1*, were defective for the use of ferric chloride and hemin in culture at neutral pH.

The poor growth of the *hap* mutants on hemin as the sole iron source may be due to expression defects in specific hemin uptake and/or processing functions, or in interacting metabolic processes. In the latter case, we noted both the *hap3Δ* and *hapXΔ* mutants had reduced transcript levels for ergosterol biosynthesis enzymes when grown in low-iron medium. A complex interaction between heme and ergosterol biosythesis/uptake has been described in *S. cerevisiae* [38]. Specifically, heme is a co-factor for some ergosterol biosynthetic enzymes and mutations in genes for heme biosynthesis (i.e., *HEM3* and *HEM4*) contribute to suppression of the sterol auxotrophy seen in *erg25* mutants. The *hem3* and *hem4* mutations allow yeast to take up exogenous sterols under aerobic conditions. We speculate that a similar interplay between heme and ergosterol biosynthesis may occur in *C. neoformans*, although additional explanations are possible given that Hap3 and HapX regulate other heme-dependent functions including electron transport. Interestingly, the *hap3Δ* and *hap5Δ* mutants were more tolerant of fluconazole on iron-rich medium, but not on low-iron medium, compared with the *hapXΔ* mutant (data not shown). This result is consistent with an interaction between iron metabolism and fluconazole inhibition of ergosterol biosynthesis. We previously found that a defect in iron uptake due to a *cfo1Δ* mutation caused an increased sensitivity to fluconazole that could be remediated by exogenous heme [18]. However, in the case of the *hap* mutants, the results presented here suggest more complex interactions between growth temperature, heme, and ergosterol biosynthesis that require further investigation.

The *hap3Δ* and *hap5Δ* mutants (but not the *hapXΔ* mutant) displayed a smaller polysaccharide capsule that is a major virulence factor, as well as an interesting phenotype of better growth at 37°C, when compared with the *hapXΔ* mutant or the WT strain. The growth phenotype may reflect a derepression of functions to deal with stressful conditions such as elevated temperature. In support of this idea, it has been shown that the CCAAT-binding complex coordinates the response to oxidative stress in *A. nidulans* [39]. We also found that hemin improved the growth of all strains at 37°C. This observation is intriguing, and may indicate that hemin acts both as an iron source and as a signal to influence the response to elevated temperature and other stresses. Overall, this analysis of *hap3Δ* and *hap5Δ* mutants revealed a new aspect of the response to heme in *C. neoformans* and additional work is clearly needed to examine the role of heme in the context of adaptation to host temperature.

Despite a reduced capsule size, the *hap3Δ* mutant did not show a virulence defect when compared with the WT strain. In contrast, the *hapXΔ* mutant showed a modest virulence defect with a delay in mortality of 3–5 days relative to WT. Previously, we observed that the *cft1Δ* and *cfo1Δ* mutants, with defects in the high-affinity, reductive iron uptake system, were still able to cause disease in mice, although at an attenuated level. We hypothesized that these mutants were likely to be defective for transferrin use *in vivo* (based on their phenotypes in culture), but their ability to grow and eventually cause disease in mice might result from heme utilization. In this context, we anticipated that examining the virulence of *hap3Δ cfo1Δ* and *hapXΔ cfo1Δ* double mutants would provide further insights because these mutants were unable to utilize ferric chloride or hemin as the sole iron sources *in vitro*. Although the mutants were attenuated for virulence relative to the WT strain, they showed equivalent virulence to the *cfo1Δ* single mutant and an additive contribution of the mutations was not observed. It is possible that heme utilization is not required for virulence in *C. neoformans*, that the mutants are only partially debilitated for hemin use or that there are mechanisms other than heme utilization that explain the disease seen in mice infected with the *cft1Δ* or *cfo1Δ* single mutants. We favor the explanation that there are redundant iron acquisition and heme utilization mechanisms, and that some of these may be uniquely expressed when the fungus is in the host environment. In addition, the defect in growth on hemin as a sole iron source might arise from interactions between heme and ergosterol biosynthesis, as mentioned above.

Candidate alternate pathways for iron acquisition could include the low-affinity iron uptake system identified by Jacobson et al. [37] as well as novel heme acquisitions systems involving extracellular hemophores such as those identified in bacterial pathogens [40]. It is also likely that there are differences between growth on solid and liquid medium, and host tissue, that influence iron acquisition. One *in vivo* condition that is particularly relevant is the pH arising from pathogen metabolism at sites of infection or in localized host niches such as the phagolysosome [36], [41]. Under the acidic condition, the ferrous form of iron would be predominant, which may bypass reductive iron uptake systems and may be transported by some other uptake pathway, or even non-specifically. Indeed, we found that acidification of the growth medium rescued the growth of the *hap3Δ cfo1Δ*, *hap5Δ cfo1Δ* and *hapXΔ cfo1Δ* double mutants on ferric chloride and hemin.

### The influence of Hap3 and HapX on transcription in *C. neoformans* and other fungi

Our microarray experiments further characterized the roles of the Hap3 and HapX proteins in the response to low-iron conditions and different iron sources. This work revealed that HapX influenced the transcript levels of 2041 genes in response to iron deprivation, and that Hap3 had a much more limited role (313 genes). The differentially expressed genes in the *hap3Δ* and *hapXΔ* mutants mainly overlapped in functions related to ATP synthesis-coupled electron transport, oxidative phosphorylation and mitochondrial electron transport. The regulation of iron-dependent functions such as TCA cycle and respiration components during iron limitation appears to be a conserved function of HapX and related orthologs in other fungi. For example, microarray analysis in *S. pombe* revealed that the HapX ortholog Php4 regulated the expression of 86 genes in response to iron starvation [25]. Specifically, Php4 was found to negatively regulate the transcription of iron-dependent functions under low-iron conditions. One of these genes encoded the iron repressor Fep1 and others encoded iron-requiring functions for the TCA cycle, the electron transport chain, amino acid biosynthesis and Fe-S cluster biosynthesis. In addition, Php4 mediated down-regulation of genes for the response to oxidative stress. These results indicate a primarily negative regulatory role for Php4, although it is possible that examination of Php4 function under other conditions may reveal positive regulatory contributions. In *A. nidulans*, HapX and the CCAAT-binding complex also repress the expression of genes encoding proteins in iron-dependent pathways and in heme biosynthesis [24]. However, the proteome analysis of Hortschansky et al., [24] additionally revealed that loss of HapX results in lower levels of proteins with a variety of other functions, including metabolic and proteolytic enzymes. From our microarray data, it appears that the *C. neoformans* HapX protein has a similar negative regulatory influence on iron-dependent pathways (e.g., cytochromes) as Php4 and HapX in *S. pombe* and *A. nidulans*. However, we also found that the *C. neoformans* protein has a positive influence on gene expression including siderophore transport functions. Overall, further comparative studies are needed to investigate the sets of genes that are positively regulated by the HapX proteins in the different fungi.

### HapX and Cir1 have shared and distinct influences on iron uptake functions

The microarray data revealed that HapX and Cir1 shared GO terms for iron ion transport and siderophore transport under low-iron conditions, and that HapX makes little contribution to the regulation of genes for high-affinity iron uptake (i.e., *CFT1* and *CFO1*). No other shared GO categories for HapX and Cir1 were identified under any of the iron-replete conditions. Thus, HapX and Cir1 may partner in the regulation of genes for iron acquisition related to siderophore transport. It is thought that *C. neoformans* does not make its own siderophores and instead employs transporters to steal siderophores from other microbes [14], [37]. Dissecting the roles of the siderophore transporters in *C. neoformans* will be challenging given that there are at least seven candidate genes and the corresponding proteins may have overlapping transport specificities [21]. Further studies of the siderophore utilization by the *hapXΔ* mutant may shed light on uptake and provide support for or against the emerging view that HapX regulation of iron uptake functions may primarily be important during *C. neoformans* growth in the environment rather than in mammalian hosts. This idea is consistent with the modest attenuation of virulence seen with the *hapXΔ* mutant; the partial contribution to disease may result from positive and/or negative regulatory influences of HapX on other genes, coupled with possible redundancy with Cir1 and other regulators. Certainly, it is likely that Cir1 and other regulators participate in adapting iron-related metabolism to the host environment.

The absence of a role for siderophore transport in the virulence of *C. neoformans* would be consistent with observations in the fungal pathogen *Aspergillus fumigatus* [14], [42], [43]. This fungus relies on siderophore production and uptake to acquire iron and cause disease in a mammalian host [42], [43]. Reductive iron uptake is not required for virulence, in contrast to the situation in *C. neoformans* [17], [42]. The iron-regulatory GATA transcription factor SreA, which has sequence similarity to Cir1, regulates the expression of genes for siderophore biosynthesis but, interestingly, loss of SreA does not attenuate virulence [44]. This result suggest that other regulatory mechanisms, perhaps including a response to iron deprivation mediated by a HapX ortholog, may contribute to siderophore biosynthesis during infection.

### An emerging iron regulatory network for host and environmental iron acquisition

Previous work on the major iron regulator Cir1 revealed that the protein influences the expression of a large number of transcription factors [15]. In addition, several groups have identified other transcriptional regulators (e.g., Nrg1, Sre1, Tup1, and Rim101) that influence the expression of iron uptake genes [27], [31]–[34]. Interactions between Cir1 and other factors likely comprise a regulatory network to control iron acquisition during cryptococcal growth in the environment and in the host. In addition, Cir1 and these transcriptional regulators may regulate one another to fine tune the expression of key iron uptake functions. Our microarray data and follow up quantitative RT-PCR studies support this view because HapX positively regulates *CIR1* transcript levels under low-iron conditions. Given the possibility that HapX plays a role in environmental iron acquisition via siderophore transport, we propose that the different transcription factors function in an interconnected iron regulatory network to regulate specific iron uptake functions. Thus, we hypothesize that HapX would regulate functions for environmental iron uptake and Cir1 would control these functions in partnership with HapX; Cir1 would additionally regulate functions for iron acquisition in the host environment. Rim101 is a likely additional partner because O'Meara et al. [27] found that this factor regulates the expression of numerous iron uptake functions in *C. neoformans* including those expected to play roles in the environment (siderophore transporters) and those that function in the host (e.g., high-affinity iron uptake). Rim101 may also participate in the pH response and therefore have a more general role in iron acquisition, along with Cir1. Interestingly, O'Meara et al. [27] and Liu et al. [28] did not find a virulence defect for the *rim101* mutant and the protein may therefore primarily influence environmental iron acquisition. As additional regulatory connections are evaluated, it may be possible to include other regulators that influence iron uptake functions in the model. In particular, the sterol regulatory factor, Sre1, that mediates the *C. neoformans* response to hypoxia, may make a contribution during infection [33], [34].

### HapX in *C. neoformans* has a novel positive influence on iron regulation

We found that HapX positively regulates the transcript level of the *C. neoformans* GATA factor Cir1 under low and high iron conditions, and that Cir1 has little influence on the transcript levels of *HAPX*. These results are strikingly different from the negative regulatory feedback relationships found for the corresponding HapX-related proteins and GATA-type iron regulatory proteins in *A. nidulans*, *C. albicans* and *S. pombe*. For example, HapX represses the expression of *sreA* under the low-iron condition and binds with the CCAAT-binding complex proteins to the *sreA* promoter in *A. nidulans* [24]. Similarly, SreA binds to a GATA element in the promoter of the *hapX* gene and deletion of *sreA* results in elevated *hapX* transcripts under iron-replete conditions. The coupled relationship is further reinforced by the observation that an *sreA hapX* double mutant is synthetically lethal. In *S. pombe*, Php4 (the HapX ortholog) negatively regulates the transcript level of the GATA iron-responsive repressor *fep1+* under low-iron conditions and, as in *A. nidulans*, the reciprocal repression of *php4+* transcription by Fep1 under iron-replete conditions is also seen [23], [25], [26]. Finally, in *Candida albicans*, Lan et al. [45] found that the transcript for the *HAPX* ortholog, *HAP43*, is elevated upon iron starvation, and it is repressed by the GATA factor Sfu1 under iron-replete conditions. Overall, our results highlight a major difference between the role of HapX in *C. neoformans* compared with its role in *A. nidulans* and *S. pombe*. That is, HapX in *C. neoformans* has a positive regulatory influence on Cir1, compared with the negative influence of the corresponding proteins on the GATA factor genes *sreA* and *fep1+* in *A. nidulans* and *S. pombe*, respectively. In addition, we note that HapX in *C. neoformans* has both positive and negative regulatory roles. So far, there is evidence from proteome studies that HapX in *A. nidulans* may also have positive and negative regulatory influences [24]. For *S. pombe*, transcriptional profiling of the *php4Δ* mutant under low iron conditions indicates a primarily negative regulatory role for the expression of genes encoding iron-requiring proteins, although this does rule out potential positive role in the expression of other genes [25]. A recent analysis of HapX in *A. fumigatus* also revealed both positive and negative regulatory influences [46]. Interestingly, this study also identified a role for HapX in virulence, a finding consistent with the positive role of HapX in siderophore production. There is also evidence that the HapX-related protein Hap43 plays a positive regulatory role in response to iron starvation in *C. albicans* [47]. The insights reported here into the role of HapX in *C. neoformans*, when combined with our analysis of Cir1 [15] and the recent work on Rim101 [27], emphasize the complexities of iron regulation and uptake mechanisms in *C. neoformans*, and reveal differences in regulatory strategies between fungi.

## Materials and Methods

### Ethics statement

This study was carried out in strict accordance with the guidelines of the Canadian Council on Animal Care. The protocol for the virulence assays employing mice (protocol A08-0586) was approved by the University of British Columbia Committee on Animal Care.

### Strains and growth conditions

The *C. neoformans* variety *grubii* strains (serotype A, MATα) used in this study are listed in Table S10 of the Supplemental Information. The *C. neoformans* Genome ORF Knockout Collection Version 1.0 (CnKOv1.0) was obtained from the American Type Culture Collection (ATCC) [28]. Cells were routinely grown in yeast extract, bacto-peptone medium with 2.0% glucose (YPD, Difco) or yeast nitrogen base (YNB, Difco) with 2.0% glucose. Defined low-iron medium (LIM) was prepared as described [48]. Briefly, YNB media was prepared in iron-chelated water using Chelex-100 (Invitrogen), adjusted to pH 7.0 with 3-morpholinopropanesulfonic acid (MOPS), and iron limitation was achieved with 100 µM bathophenanthroline disulfonate (BPS). The same medium was also used to test growth under acidic conditions, after adjustment to pH 5.0 with hydrochloric acid. To prepare iron-replete media, FeCl_3_, holo-transferrin, hemin or feroxamine were added to low-iron medium at the concentrations indicated in the text. Intracellular iron was depleted by pre-culturing cells in low-iron medium at 30°C for at least 16 h before cells were transferred to iron-replete media, as described previously [17], [18]. For growth assays on solid media, ten-fold serial dilutions of cells were spotted onto plates indicated in the text with or without the supplemented iron sources. Plates were incubated at 30°C for two days before being photographed.

### Construction of mutants

Gene sequences were obtained from the *C. neoformans* var. *grubii* serotype A genome database (http://www.broad.mit.edu/annotation/genome/cryptococcus_neoformans). Sequences of *Saccharomyces cerevisiae* Hap2 (NP_011277), Hap3 (NP_009532.1), Hap5 (NP_015003) and *Aspergillus nidulans* HapX (XP_681520) were used to search for *Cryptococcus* homologs of Hap2 (CNAG_07435.1), Hap3 (CNAG_02215.1), Hap5 (CNAG_07680.1) and HapX (CNAG_01242.1), respectively. Deletion mutants for *HAP3*, *HAP5* and *HAPX* were constructed by homologous recombination using gene specific knock-out cassettes, which were amplified by three-step overlapping PCR with primers listed in Supplemental Information (Table S11). To construct the *hap3Δ* mutant, a gene-specific disruption cassette was constructed by PCR using primers H9Hap3.1-KO1, H9Hap3.1-KO2, H9Hap3.1-KO3, H9Hap3.1-KO4, H9Hap3.1-KO5 and H9Hap3.1-KO6, with genomic DNA and the plasmid pCH233 as templates [49], [50]. The amplified construct was introduced into the wild-type strain by biolistic transformation, as described [51]. The genomic region of 431 bp of the coding sequence of *HAP3* was replaced with the nourseothricin acetyltransferase gene (*NAT*) using 5′ and 3′ flanking sequences of *HAP3*. Positive transformants were identified by PCR and confirmed by Southern blot analysis (Figure S4). For construction of the reconstituted strain, the *HAP3* gene was amplified by PCR using wild-type genomic DNA and primers Hap3.1_Re.F and Hap3.1_Re.R. The amplified 2.6 Kb DNA fragment containing wild type *HAP3* was digested with KpnI/SpeI and ligated with pJAF1 to construct pWH106 containing the neomycin resistance marker (*NEO*). The plasmid was digested with NdeI and introduced into the *hap3Δ* mutant.

To construct the *hap5Δ* mutant, a gene-specific disruption cassette was prepared by PCR using primers H9Hap5-KO1, H9Hap5-KO2, H9Hap5-KO33, H9Hap5-KO34, H9Hap5-KO5 and H9Hap5-KO36, with genomic DNA and the plasmid pCH233 as templates. The amplified construct was introduced into the wild-type strain by biolistic transformation. The genomic region of 690 bp of the coding sequence of *HAP5* was replaced with the nourseothricin acetyltransferase gene (*NAT*) using 5′ and 3′ flanking sequences of *HAP5*. Positive transformants were identified by PCR and confirmed by Southern blot analysis (Figure S4). For construction of the reconstituted strain, the *HAP5* gene was amplified by PCR using wild-type genomic DNA and primers Hap5_Re.F and Hap5_Re.R. The amplified 3.6 Kb DNA fragment containing wild type *HAP5* was digested with KpnI/SpeI and ligated with pJAF1 to construct pWH108 containing the neomycin resistance marker (*NEO*). The plasmid was digested with XhoI and introduced into the *hap5Δ* mutant.

To construct the *hapXΔ* mutant, a gene-specific disruption cassette was constructed by PCR using primers H9HapX-KO1, H9HapX-KO2, H9HapX-KO3, H9HapX-KO4, H9HapX-KO5 and H9HapX-KO6, with genomic DNA and the plasmid pCH233 as templates. The amplified construct was introduced into the wild-type strain by biolistic transformation. The genomic region of 2474 bp of the coding sequence of *HAPX* was replaced with the nourseothricin acetyltransferase gene (*NAT*) using 5′ and 3′ flanking sequences of *HAPX*. Positive transformants were identified by PCR and confirmed by Southern blot analysis (Figure S4). For construction of the reconstituted strain, the *HAPX* gene was amplified by PCR using wild-type genomic DNA and primers HapX_Re.F and HapX_Re.R. The amplified 4.0 Kb DNA fragment containing wild type *HAPX* was digested with KpnI/SpeI and ligated with pJAF1 to construct pWH107 containing the neomycin resistance marker (*NEO*). The plasmid was digested with BlpI and introduced into the *hapXΔ* mutant.

The *CFO1* knock-out construct, which contains a neomycin resistant cassette and ∼1 kb upstream and downstream DNA sequences of the *CFO1* gene, was amplified using primer pair (CFO1-KO5 and -KO6) and genomic DNA from strain WK3-4 (*cfo1Δ cfo2Δ* double knock-out mutant) [18]. The PCR product was introduced into mutant strains (*hap3Δ*, *hap5Δ* or *hapXΔ*) by biolistic transformation. Transformants were screened by colony-PCR with primer pair CFO1-KO1 and NATstart. Four independent *hap3Δ cfo1Δ* mutants, five *hap5Δ cfo1Δ* and four *hapXΔ cfo1Δ* mutant strains were generated. Two strains from each double knock-out mutant, namely *hap3Δ cfo1Δ#1*, *hap3Δ cfo1Δ#2*, *hap5Δ cfo1Δ#54*, *hap5Δ cfo1Δ#61*, *hapXΔ cfo1Δ#1* and *hapX cfo1Δ#2* were studied further.

### RNA hybridization and microarray experiments

The WT strain and the *hap3Δ*, *hapXΔ*, and *cir1Δ* mutants were used for microarray analysis. Three biological replicates for each strain were grown in 25 ml of YPD overnight at 30°C. Cells were washed twice with LIM followed by growth in LIM at 30°C for an additional 16 h in order to eliminate any iron carryover from the rich medium. Cultures were then harvested and washed twice with LIM. The cells were counted and transferred to 50 ml LIM (C), LIM+100 µM of FeCl_3_ (F), LIM+5 µM of Transferrin (T) or LIM+10 µM of Hemin (H) (final density of 1.0×10^7^ cells/ml). The cells were then grown at 30°C for another 6 h and harvested for RNA extractions. RNA was purified with the RNeasy kit (Qiagen) and treated with DNase (Qiagen) following the manufacturer's recommendations. The quality of RNA was analyzed with an Agilent 2100 Bioanalyzer and cDNA was synthesized from 5 µg of total RNA by SuperScriptII Reverse Transcript Enzyme (Invitrogen). The 3DNA Array 350 kit (Genisphere) was used to label cDNA with Cy3 or Cy5 for hybridization to 70-mer microarrays (version 2.0, http://genomeold.wustl.edu/activity/ma/cneoformans/index.cgi).

### Modification of the gene list of microarray probes

Because the gene lists of microarray probes were originally designed for *C. neoformans* var. *neoformans* JEC21, they were modified for *C. neoformans* var. *grubii* H99 as described below. First, a list of 70-mer probes designed from JEC21 cDNA sequences were matched against the H99 transcriptome (using blastN algorithm in stand alone mode). Only the highest scoring hit was considered for each query sequence. Blast results were then further filtered to include only those hits that matched 67 or more nucleotides of the 70-mer probe (95.6% oligonucleotides matching). This is a conservative application of stringency criteria given the 85–90% genome sequence similarity between serotype D strain JEC21 and serotype A strain H99. An intermediate level of match stringency was selected to allow for detection of transcripts using probes from a heterologous serotype. Using the 67/70 cut-off, we obtain 4523 (4348 unique) matching H99 genes. A list of 70-mer probes designed from H99 cDNA sequences was then blasted (blastN algorithm) against the H99 transcriptome to identify matching transcripts. Again, only the highest scoring hit was considered for each query sequence. Blast results were further filtered to include only those hits with 100% matching oligonucleotides (70/70). This yielded 2485 (2447 unique) H99 genes. A comparison of the lists of H99 hits obtained from the blasts of the heterologous (JEC21) and homologous probe sequences indicated 630 genes in common. This method identified a total of 6165 matching H99 genes (Table S1). Inclusion of mitochondrial transcripts in the H99 transcriptome database only identified one additional gene - CNAG_9009 (cytochrome c-oxidase) in the BLAST (69/70 match, e-value = 2e-31).

### Microarray hybridization, statistical analysis and data mining

The following experimental design was adopted for the study: within each strain, each treatment pair (low-iron control (C) versus ferric chloride (F), transferrin (T) or hemin (H); six pairs) were hybridized to an array; and within each treatment, each strain comparison (WT versus *hap3Δ* (H3), *hapXΔ* (HX) or *cir1Δ* (C1), 6 pairs) was hybridized to an array for a total of 48 microarrays. Each strain/treatment combination was labeled an equal number of times with Cy3 and Cy5 to ensure dye balance. After hybridization, each array was scanned using the Perkin Elmer Scan Array Express. Each channel was background corrected by subtracting the lowest 10% of foreground signal intensities within each subgrid of the array. The two channels of each array were normalized to each other by Huber's variance stabilization algorithm [52]. A linear mixed effects model was applied to the normalized data in each channel. A fixed effect was included for each array, for the dye by gene interaction and for each treatment/strain combination. Random effects were included for within-array variability (each gene appeared twice on each array), technical variability (replicate culture was hybridized twice) and biological variability (48 replicate cultures were employed). The pairwise changes between treatments within strain and the pairwise changes between strains within treatment were estimated with standard errors and *p*-values based Student *t* statistics; *q*-values were computed to adjust for the false discovery rate.

For clustering, the genes that were more than 2-fold differentially expressed with a significant *q*-value (*q*<0.05) in at least one comparison were selected and the log fold change were clustered using DIANA (a divisive hierarchical clustering algorithm) [53] and visualized by heatmaps and dendrograms. All analyses were performed using the R language and environment for statistical computing [54]. The microarray data mining tool ermineJ was used to analyze the microarray dataset based on GO terms [35]. The *p*-values from the microarray data were used as input scores and gene score resampling analysis (GSA) was applied. The microarray data have been deposited in the Gene Expression Omnibus database (www.ncbi.nlm.nih.gov/geo/) under the accession number GSE22988.

### Virulence assays

The virulence of each cryptococcal strain was examined using female BALB/c mice (4 to 6 weeks old) from Charles River Laboratories (Ontario, Canada). Fungal cells were cultured in 5 ml of YPD at 30°C overnight, washed twice with PBS (Invitrogen), and resuspended in PBS. The BALB/c mice, in groups of 10, were anesthetized intraperitoneally with ketamine (80 mg/kg of body weight) and xylazine (5.5 mg/kg) in PBS and suspended on a silk thread by the superior incisors. A suspension of 5×10^4^ cells in 50 µl was slowly dripped into the nares of the anesthetized mice, and the mice were suspended for 10 minutes on the thread. The health status of the mice was monitored daily post-inoculation. Mice reaching the humane endpoint were euthanized by CO_2_ anoxia. Statistical analyses of survival differences were performed by log rank tests using GraphPad Prism 4 for Windows (GraphPad Software, San Diego, CA).

### Capsule formation

Low-iron medium was used to examine capsule formation. A single colony from a YPD plate for each strain was cultured overnight at 30°C in liquid YPD medium. Cells were harvested and diluted in low-iron water, and 10^4^ cells were added into 3 ml of LIM for further incubation at 30°C for 48 h. After incubation, the capsule was stained with India ink and examined by differential interference microscopy.

### Carbon source utilization

YNB agar was supplemented with one of the following carbon sources: 2% glucose, 2% sucrose, 2% sodium acetate, or 2% ethanol. Exponentially growing cultures were washed, re-suspended in water and adjusted to a concentration of 2×10^4^ cells per µl. The cell suspensions were diluted 10-fold serially and 5 µl of each dilution was spotted onto the plates. Plates were incubated for 2–3 days at 30°C and photographed.

### Quantitative real-time RT-PCR

Primers for real-time RT-PCR were designed using Primer Express software 3.0 (Applied Biosystems) and are listed in Table S11 of the Supplemental Information. cDNA was synthesized from the same total RNA that was used for microarray analysis using Verso Reverse Transcriptase (Thermo Scientific). PCR reactions were monitored as described previously [14], and relative gene expression was quantified based on the 2^−ΔΔ*CT*^ method [55]. 18S rRNA was used as an internal control for normalization.

## Supporting Information

Figure S1 Growth of candidate hap mutants on different iron sources. Ten-fold serial dilutions of cells (starting at 10^4^ cells) were spotted onto solid YPD medium, low-iron medium (YNB+100 µM BPS), and low-iron medium supplemented with 100 µM FeCl_3_ or 10 µM Hemin. The strains were obtained from the deletion collection of Liu et al. [28]. The gene identifications are as follows: *HAP1* (CNAG_06818), *HAP3-1* (CNAG_02215), *HAP3-2* (CNAG_01201), *HAP5* (CNAG_07680) and *HAPX* (CNAG_01242).
(0.29 MB TIF)

Figure S2 Growth of the *hap3Δ* and *hapXΔ* mutants in liquid low-iron media supplemented with ferric chloride, hemin or transferrin. The densities of liquid cultures for the WT and mutant strains was monitored at OD600 during incubation in low-iron medium or low-iron medium supplemented with 100 µM FeCl_3_, 5 µM Transferrin or 10 µM Hemin. These growth conditions and the 6 h time point were employed to prepare cells for RNA extractions and microarray analysis. Cells of the *cir1Δ* mutant were prepared under identical conditions and this mutant also shows poor growth with hemin as the sole iron source (data not shown).
(0.24 MB TIF)

Figure S3 Amino acid alignments of the *HAP* genes of *C. neoformans* with orthologs from *Saccharomyces cerevisiae* or *Aspergillus nidulans*. The amino acid sequences for Hap2 (NP_011277), Hap3 (NP_009532.1) and Hap5 (NP_015003) from *S. cerevisiae* were employed in alignments with the Hap2 (CNAG_07435), Hap3 (CNAG_02215), and Hap5 (CNAG_07680) sequences from *C. neoformans*. The HapX (CNAG_01242) sequence was aligned with the *Aspergillus nidulans* HapX (XP_681520) ortholog as previously identified [24]. The alignments were performed with T-Coffee [56].
(3.34 MB TIF)

Figure S4 Construction of *hap* deletion mutations and confirmation of mutant genotypes by genomic hybridization. Diagrams are presented for the WT loci for *HAP3* (A), *HAP5* (B) and *HAPX* (C) as well as the deletion alleles in which the nourseothricin resistance gene was used to replace the open reading frame of each gene. D) Genomic hybridization results with the probes indicated in panels A–C.
(0.16 MB TIF)

Figure S5 Venn diagrams of the numbers of shared and distinct genes regulated by HapX, Hap3 and Cir1 under different iron conditions. Venn diagram representing the numbers and overlap of differentially expressed genes (at least 2-fold) with statistical significance (*q*<0.05) in the mutants versus WT in response to different iron sources. Numbers in parentheses indicate the total for that particular treatment.
(0.30 MB TIF)

Table S1 Modified gene list for *C. neoformans* var. *grubii* H99.
(0.85 MB XLS)

Table S2 Differentially expressed genes (at least 2-fold) with statistical significance (*q* value less than 0.05) in the mutants versus wild-type in response to low-iron.
(0.39 MB XLS)

Table S3 Differentially expressed genes (at least 2-fold) with statistical significance (*q* value less than 0.05) in the mutants versus wild-type in response to ferric chloride.
(0.17 MB XLS)

Table S4 Differentially expressed genes (at least 2-fold) with statistical significance (*q* value less than 0.05) in the mutants versus wild-type in response to hemin.
(0.24 MB XLS)

Table S5 Differentially expressed genes (at least 2-fold) with statistical significance (*q* value less than 0.05) in the mutants versus wild-type in response to transferrin.
(0.28 MB XLS)

Table S6 Gene Ontology terms and the corresponding *p* values identified for each comparison using the data mining tool ermineJ.
(0.03 MB XLS)

Table S7 List of genes and their individual *p* values under each gene ontology term.
(0.13 MB XLS)

Table S8 Expression profiles of genes in the ergosterol biosynthesis pathway.
(0.03 MB XLS)

Table S9 Expression profiles of genes in the heme biosynthesis pathway.
(0.03 MB XLS)

Table S10 List of strains.
(0.04 MB DOC)

Table S11 List of oligonucleotide primers employed for strain construction and quantitative RT-PCR.
(0.03 MB XLS)
