# Supplementary figures and images for: HapX Positively and Negatively Regulates the Transcriptional Response to Iron Deprivation in Cryptococcus neoformans
Source: PLoS Pathog. 2010 Nov 24;6(11):e1001209. doi: 10.1371/journal.ppat.1001209 (PMC2991262; doi:10.1371/journal.ppat.1001209)

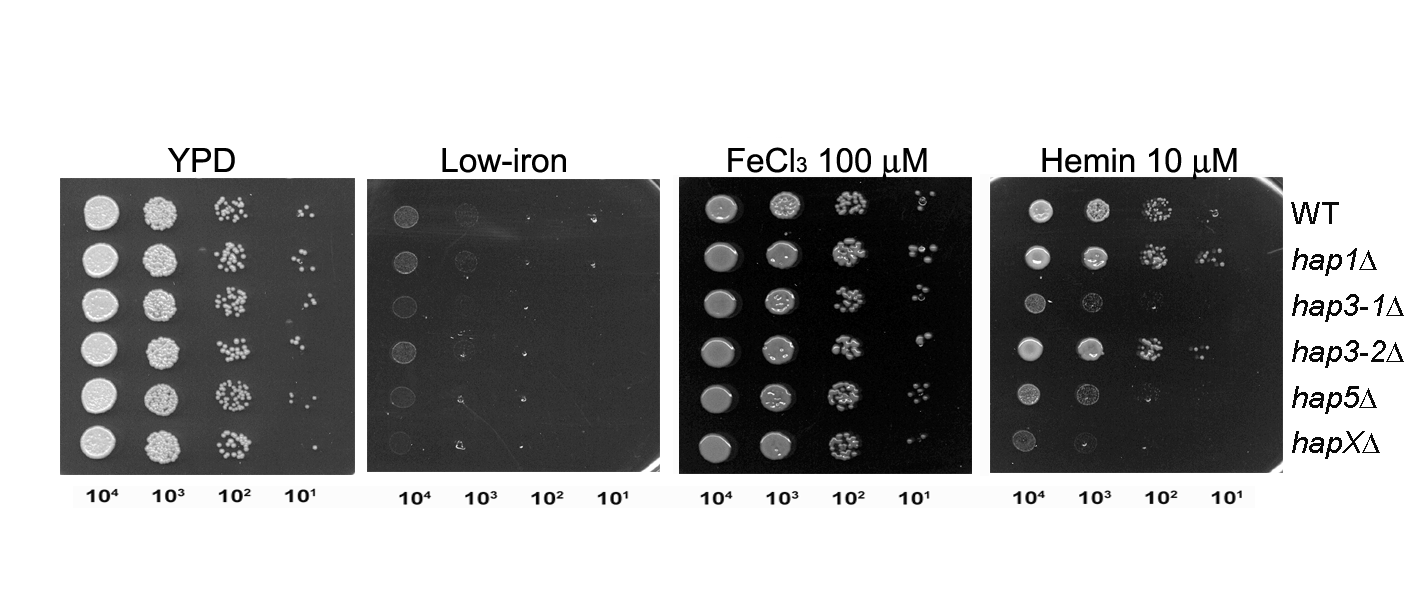

Supplement: Figure S1 — Growth of candidate hap mutants on different iron sources. Ten-fold serial dilutions of cells (starting at 104 cells) were spotted onto solid YPD medium, low-iron medium (YNB+100 µM BPS), and low-iron medium supplemented with 100 µM FeCl3 or 10 µM Hemin. The strains were obtained from the deletion collection of Liu et al. [28]. The gene identifications are as follows: HAP1 (CNAG_06818), HAP3-1 (CNAG_02215), HAP3-2 (CNAG_01201), HAP5 (CNAG_07680) and HAPX (CNAG_01242). (0.29 MB TIF) [file ppat.1001209.s001.tif]

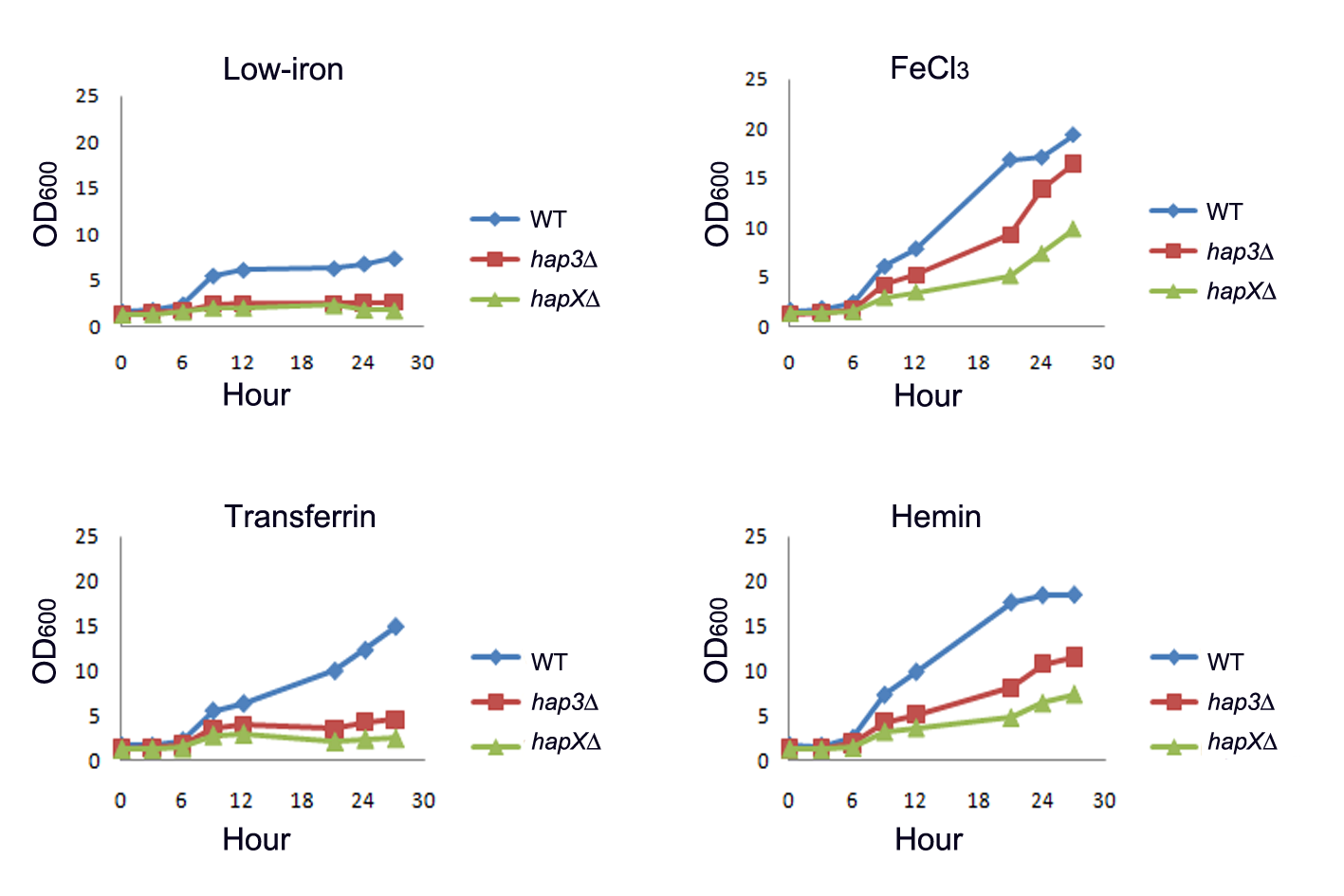

Supplement: Figure S2 — Growth of the hap3Δ and hapXΔ mutants in liquid low-iron media supplemented with ferric chloride, hemin or transferrin. The densities of liquid cultures for the WT and mutant strains was monitored at OD600 during incubation in low-iron medium or low-iron medium supplemented with 100 µM FeCl3, 5 µM Transferrin or 10 µM Hemin. These growth conditions and the 6 h time point were employed to prepare cells for RNA extractions and microarray analysis. Cells of the cir1Δ mutant were prepared under identical conditions and this mutant also shows poor growth with hemin as the sole iron source (data not shown). (0.24 MB TIF) [file ppat.1001209.s002.tif]

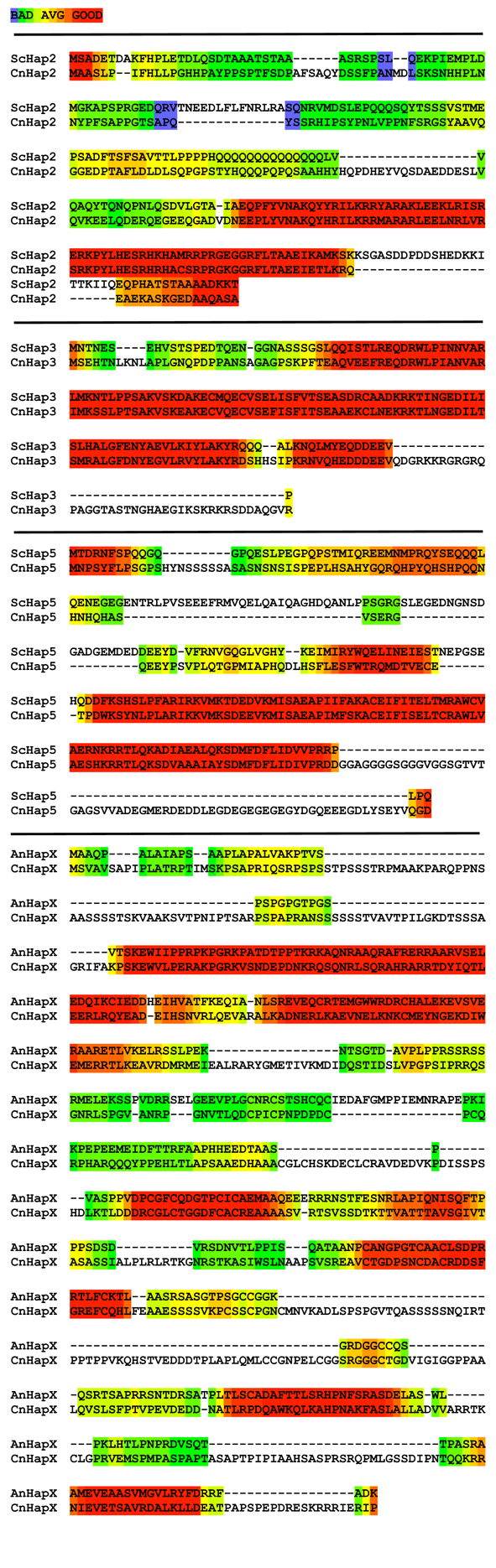

Supplement: Figure S3 — Amino acid alignments of the HAP genes of C. neoformans with orthologs from Saccharomyces cerevisiae or Aspergillus nidulans. The amino acid sequences for Hap2 (NP_011277), Hap3 (NP_009532.1) and Hap5 (NP_015003) from S. cerevisiae were employed in alignments with the Hap2 (CNAG_07435), Hap3 (CNAG_02215), and Hap5 (CNAG_07680) sequences from C. neoformans. The HapX (CNAG_01242) sequence was aligned with the Aspergillus nidulans HapX (XP_681520) ortholog as previously identified [24]. The alignments were performed with T-Coffee [56]. (3.34 MB TIF) [file ppat.1001209.s003.tif]

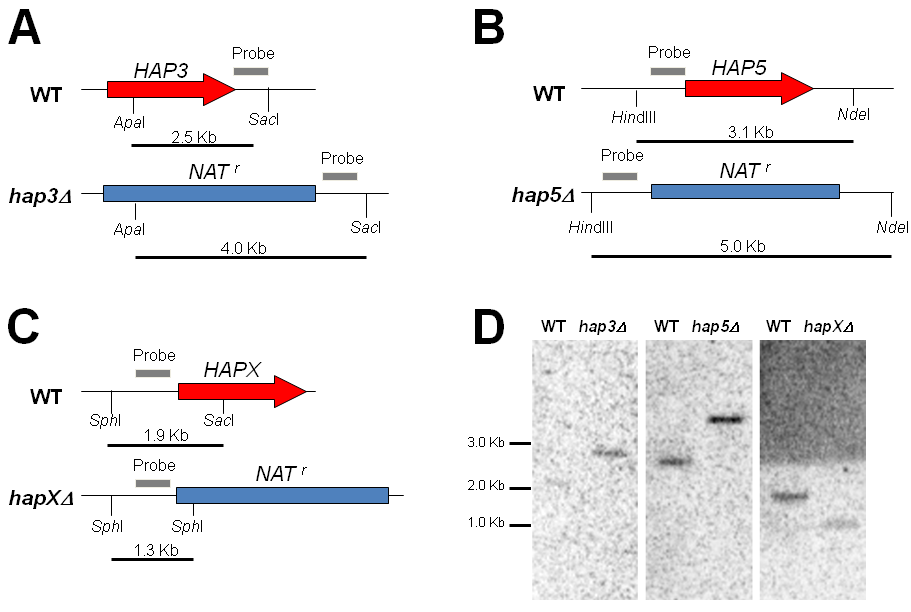

Supplement: Figure S4 — Construction of hap deletion mutations and confirmation of mutant genotypes by genomic hybridization. Diagrams are presented for the WT loci for HAP3 (A), HAP5 (B) and HAPX (C) as well as the deletion alleles in which the nourseothricin resistance gene was used to replace the open reading frame of each gene. D) Genomic hybridization results with the probes indicated in panels A–C. (0.16 MB TIF) [file ppat.1001209.s004.tif]

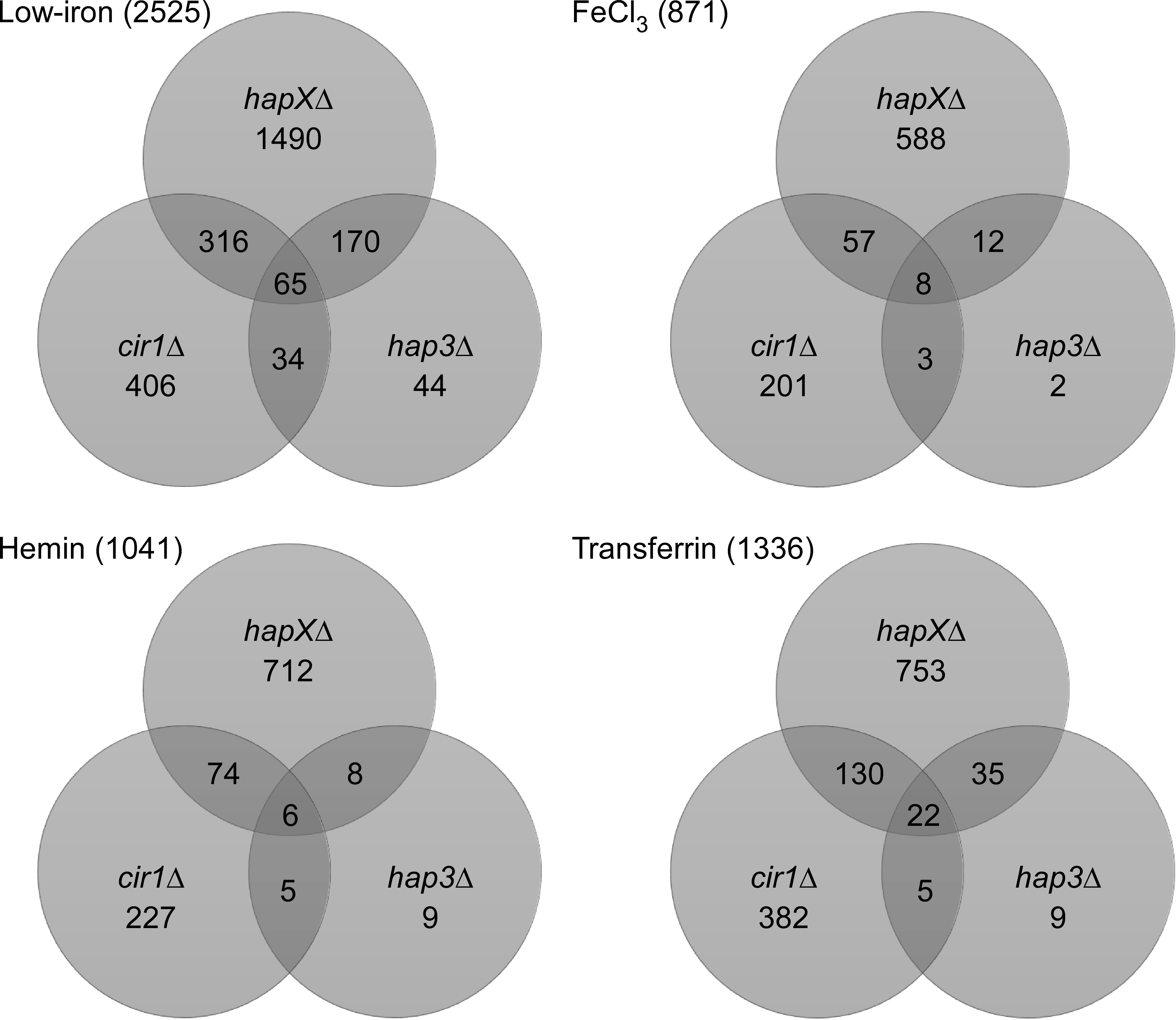

Supplement: Figure S5 — Venn diagrams of the numbers of shared and distinct genes regulated by HapX, Hap3 and Cir1 under different iron conditions. Venn diagram representing the numbers and overlap of differentially expressed genes (at least 2-fold) with statistical significance (q<0.05) in the mutants versus WT in response to different iron sources. Numbers in parentheses indicate the total for that particular treatment. (0.30 MB TIF) [file ppat.1001209.s005.tif]
