# Supplementary material for: HapX Positively and Negatively Regulates the Transcriptional Response to Iron Deprivation in Cryptococcus neoformans
Source: PLoS Pathog. 2010 Nov 24;6(11):e1001209. doi: 10.1371/journal.ppat.1001209 (PMC2991262; doi:10.1371/journal.ppat.1001209)
Supplement: Table S10 — List of strains. (0.04 MB DOC) [file ppat.1001209.s015.doc]

| Strains | Genotype | References |
| --- | --- | --- |
| H99 | *MATα* | [1] |
| *hap3*Δ | *MATα hap3Δ ::NAT* | This study |
| *hap3*Δ::*HAP3* | *MATα hap3Δ ::NAT, HAP3-NEO* | This study |
| *hap5*Δ | *MATα hap5Δ ::NAT* | This study |
| *hap5*Δ::*HAP5* | *MATα hap5Δ ::NAT, HAP5-NEO* | This study |
| *hapX*Δ | *MATα hapXΔ ::NAT* | This study |
| *hapX*Δ::*HAPX* | *MATα hapXΔ ::NAT, HAPX-NEO* | This study |
| *cfo1*Δ | *MATα cfo1Δ ::NAT* | [2] |
| *hap3*Δ *cfo1*Δ | *MATα hap3Δ ::NAT, cfo1Δ ::NEO* | This study |
| *hap5*Δ *cfo1*Δ | *MATα hap5Δ ::NAT, cfo1Δ ::NEO* | This study |
| *hapX*Δ *cfo1*Δ | *MATα hapXΔ ::NAT, cfo1Δ ::NEO* | This study |

**Table S10. Strains used in this study.**

1. Perfect JR, Ketabchi N, Cox GM, Ingram CW, Beiser CL (1993) Karyotyping of *Cryptococcus* *neoformans* as an epidemiological tool. J Clin Microbiol 31: 3305-3309.

2. Jung WH, Hu G, Kuo W, Kronstad JW (2009) The role of ferroxidases in iron uptake and virulence of *Cryptococcus neoformans.* Eukaryotic Cell. 8: 1511-1520.
